# BioGraphFusion: graph knowledge embedding for biological completion and reasoning

**DOI:** 10.1093/bioinformatics/btaf408

**Published:** 2025-07-18

**Authors:** Yitong Lin, Jiaying He, Jiahe Chen, Xinnan Zhu, Jianwei Zheng, Tao Bo

**Affiliations:** College of Computer Science and Technology, Zhejiang University of Technology , 288 Liuhe Road, Xihu District, Hangzhou, Zhejiang Province, 310023, China; College of Computer Science and Technology, Zhejiang University of Technology , 288 Liuhe Road, Xihu District, Hangzhou, Zhejiang Province, 310023, China; College of Computer Science and Technology, Zhejiang University of Technology , 288 Liuhe Road, Xihu District, Hangzhou, Zhejiang Province, 310023, China; College of Computer Science and Technology, Zhejiang University of Technology , 288 Liuhe Road, Xihu District, Hangzhou, Zhejiang Province, 310023, China; College of Computer Science and Technology, Zhejiang University of Technology , 288 Liuhe Road, Xihu District, Hangzhou, Zhejiang Province, 310023, China; Key Laboratory of Endocrine Glucose & Lipids Metabolism, Department of Endocrinology, , Shandong Provincial Hospital Affiliated to Shandong First Medical University , 324 Jingwu Road, Huaiyin District, Jinan, Shandong Province, 250021, China

## Abstract

**Motivation:**

Biomedical knowledge graphs (KGs) are crucial for drug discovery and disease understanding, yet their completion and reasoning are challenging. Knowledge embedding (KE) methods capture global semantics but struggle with dynamic structural integration, while graph neural networks (GNNs) excel locally but often lack semantic understanding. Even ensemble approaches, including those leveraging language models, often fail to achieve a deep, adaptive, and synergistic co-evolution between semantic comprehension and structural learning. Addressing this critical gap in fostering continuous, reciprocal refinement between these two aspects in complex biomedical KGs is paramount.

**Results:**

We introduce BioGraphFusion, a novel framework for deeply synergistic semantic and structural learning. BioGraphFusion establishes a global semantic foundation via tensor decomposition, guiding an LSTM-driven mechanism to dynamically refine relation embeddings during graph propagation. This fosters adaptive interplay between semantic understanding and structural learning, further enhanced by query-guided subgraph construction and a hybrid scoring mechanism. Experiments across three key biomedical tasks demonstrate BioGraphFusion’s superior performance over state-of-the-art KE, GNN, and ensemble models. A case study on cutaneous malignant melanoma 1 highlights its ability to unveil biologically meaningful pathways.

**Availability and implementation:**

Source code and all data underlying this article are freely available in the GitHub repository at https://github.com/Y-TARL/BioGraphFusion.

## Graphical Abstract

**Figure:**
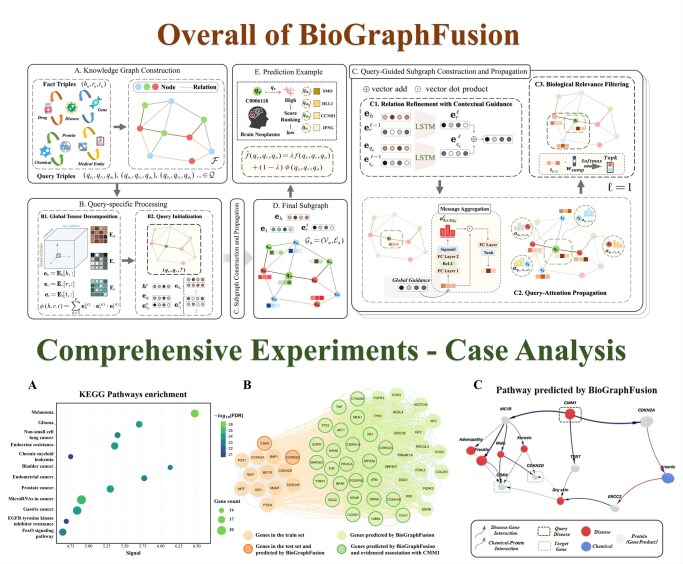

## 1 Introduction

Knowledge graphs (KGs) are semantic networks that represent relationships between entities as a set of triples (h,r,t), where *h* and *t* denote the head and tail entities, respectively, and *r* represents the relation connecting them (Wang *et al.* 2024a). These graphs model real-world concepts and their interactions through nodes (entities) and edges (relations). Specifically, biological KGs have extended this framework to encompass entities such as diseases, genes, drugs, chemicals, and proteins, facilitating a structured understanding of clinical knowledge.

Technically, large-scale biological KGs such as DisGeNET (Piñero *et al.* 2020), STITCH (Szklarczyk *et al.* 2016), and SIDER (Kuhn *et al.* 2016) are widely used in biomedical research, which support applications including disease gene prediction (Vilela *et al.* 2023), drug–target interaction (Qiao *et al.* 2024), and drug–drug correlation (Wang *et al.* 2024b).

Many such tasks demand for practical techniques of knowledge graph completion (KGC) (Chen *et al.* 2020) and knowledge graph reasoning (KGR) (Liang *et al.* 2024). Fundamentally, both techniques involve predicting the answer to a query of the form (h, r, ?) (Meng *et al.* 2024), to identify the missing tail entity. While both may be considered as link prediction, they differ: KGC primarily predicts missing direct links (entities or relations) by identifying patterns in existing graph data. Extending this, KGR is a broader task that infers complex or multi-step knowledge, often employing logical inference mechanisms, rule-based systems, or multi-hop path analysis to deduce unstated facts. Thus, KGC focuses on completing the KG based on observed patterns, while KGR derives new insights through deeper inferential processes.

For KGC and KGR, knowledge embedding (KE) and graph structure propagation (GSP) are prevalent approaches, as detailed in foundational works (Tang *et al.* 2024; Liang *et al.* 2024). KE, often termed a latent feature model (Nickel *et al.* 2016), embeds entities and relations into continuous vector spaces, capturing semantic information to score candidate entities directly. For instance, RotatE (Sun *et al.* 2019) models relations as rotations in complex space (h°r≈t), while CP-N3 (Lacroix *et al.* 2018) enhances performance by factorizing higher-order interactions. While KE techniques excel at capturing semantics, they often overlook structural patterns—such as multi-hop paths—limiting their reasoning capabilities over complex, multi-relational biomedical graphs (Liu *et al.* 2022; Peng *et al.* 2023).

In contrast, GSP borrows its main architecture from graph neural networks (GNNs) (Yu *et al.* 2021), which have significantly advanced network analysis by propagating messages between entities, thereby partially capturing topological information. Representatives such as GNN4DM (Gézsi and Antal 2024) demonstrate the power of these approaches in tasks like discovering overlapping functional disease modules through the integration of network topology and genomic data. However, these methods, while adept at structural modeling, often tend to overemphasize topological information at the expense of deeper semantic associations and the rich content of relations.

Recognizing the limitations of traditional KE and GSP methods, and with the advent of powerful pre-trained language models (LMs), more advanced approaches have emerged for KGC and KGR. These methods often seek to incorporate richer semantic understanding directly from textual data or find novel ways of integrating semantic and structural information, moving beyond the KE or GSP paradigms alone. For instance, KG-BERT (Yao *et al.* 2019) uses pre-trained LMs to score textualized triples, prioritizing semantics but with high computational costs. Similarly, LASS (Shen *et al.* 2022) attempts fusion by embedding natural language semantics with graph structure through LM fine-tuning and probabilistic reconstruction loss, yet its loss-mediated interaction limits deeply adaptive integration.

While these approaches significantly advanced semantic and structural learning for KGC and KGR, they underscore a persistent challenge: achieving deep, dynamic coupling where semantic guidance and structural propagation synergistically co-evolve. Many methods, despite innovations, still struggle with fully reciprocal, adaptive refinement between rich semantic understanding and nuanced structural learning. This critical gap—the difficulty in developing a framework that enables a mutually enhancing co-evolution between semantic and structural learning—motivates BioGraphFusion. BioGraphFusion is a novel framework designed for such a profound synergistic integration, which leverages semantic insight, primarily drawing from principles of KE for global context, and combines it with dynamic structural reasoning, inspired by GSP techniques. The overall goal is for joint optimization of node and relation embeddings, thereby addressing the limitations in achieving the deep and adaptive semantic-structural interplay seen in prior methods.

BioGraphFusion actualizes this for biomedical KGs by weaving global semantic modeling with dynamic structural reasoning. Initially, a canonical polyadic (CP) decomposition (Kolda and Bader 2009) module establishes a global semantic foundation, extracting low-dimensional embeddings capturing overarching biological associations and cross-domain interactions. This global semantic framework then actively steers structural learning. An LSTM-based gating mechanism dynamically refines relation embeddings during message propagation, adapting them to evolving semantic contexts and enabling the model to better capture long-range dependencies crucial for complex biological pathways. Further, a query-guided subgraph construction component focuses structural exploration on pertinent biological regions, ensuring message passing and representation learning concentrate on relevant interactions. Finally, a hybrid scoring mechanism orchestrates synergy between these semantic and structural representations. Such balanced integration empowers the semantic model to guide dynamic refinement of graph-based representations, fostering deep optimization of intricate edge embeddings. This process ensures a reciprocal and adaptive refinement cycle, where semantic understanding and structural learning iteratively enhance each other.

## 2 Materials and methods

### 2.1 Dataset overview and task design

To advance biological KG completion and reasoning, we introduce three tasks integrating multi-source biomedical data. First, the disease–gene association prediction task (Wang *et al.* 2024a) identifies missing disease-related genes by leveraging a primary dataset enriched with drug-disease and protein-chemical information. Second, the protein–chemical interaction task focuses on identifying compounds that interact with specific proteins, using core interaction data supplemented by auxiliary associations. Finally, the cross-medical ontology reasoning task employs the UMLS Terminology (Bodenreider 2004). This task functions as link prediction: given a head concept and relation type, the model predicts the tail concept, inferring diverse ontological relationships, including hierarchical and associative links. Detailed dataset statistics and integration protocols for all tasks are summarized in Section 1, available as supplementary data at *Bioinformatics* online.

### 2.2 Overview of BioGraphFusion

BioGraphFusion achieves high performance in biomedical completion and reasoning by fostering a deep synergistic interplay between KE and GSP principles. By incorporating global semantic knowledge from KEs to guide the graph propagation process, our proposal effectively captures both direct and long-range relationships in biomedical graphs.

**Figure 1.**
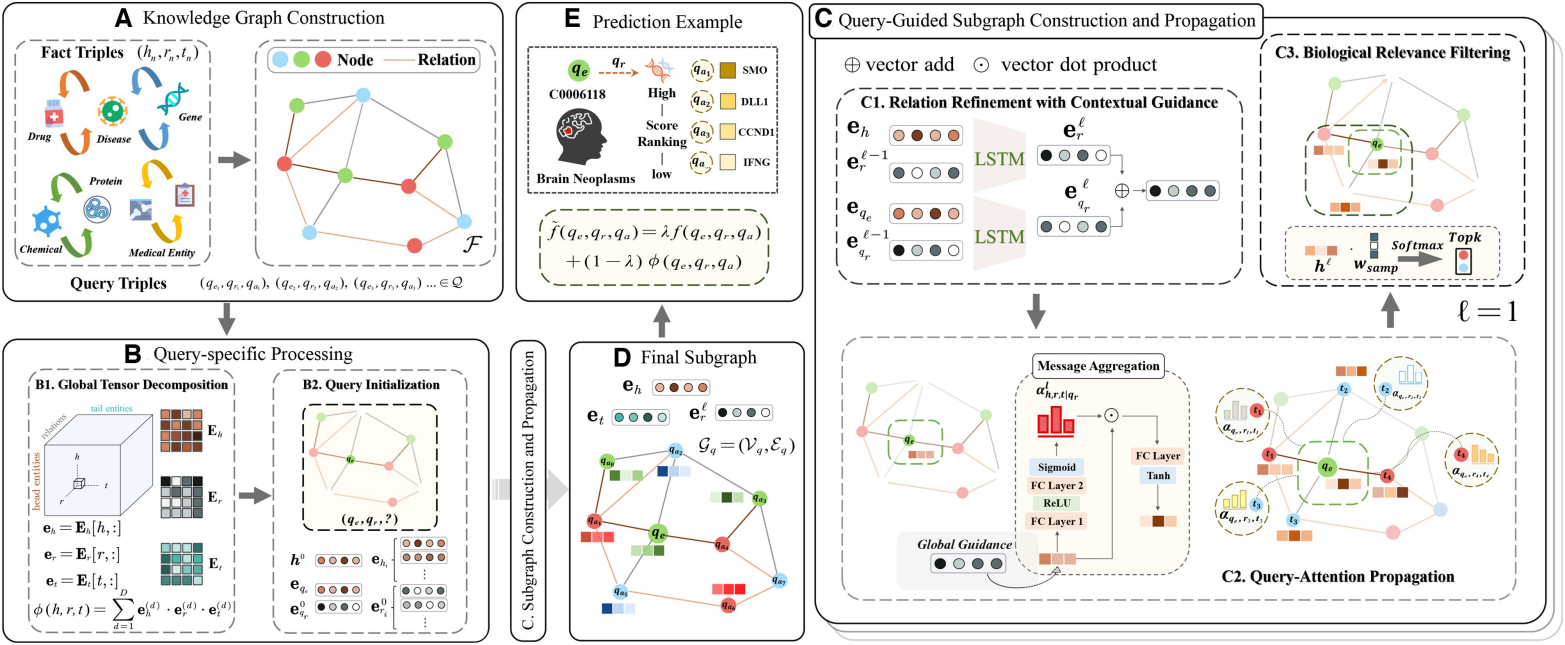
Overview of the BioGraphFusion framework. (A) Knowledge graph construction: integrating biomedical datasets to form a unified knowledge graph for downstream tasks. (B) Query-specific processing: a two-step process involving (B1) global tensor decomposition that captures latent biological associations, and (B2) query initialization that guides the guide the subsequent process. (C) Subgraph construction and propagation: iteratively builds a query-relevant subgraph through neighborhood expansion and propagation, including (C1) relation refinement via LSTM, (C2) query-attention propagation with context-based attention weights, and (C3) biological relevance filtering to select the most pertinent entities. (D) Final subgraph. (E) Scoring integration that balances structural-semantic information and Prediction Example that selects the most promising predictions, with a focus on brain neoplasms.

As illustrated in Fig. 1, BioGraphFusion comprises three key components. First, global biological tensor encoding (Section 2.2.2) employs CP decomposition to extract low-dimensional embeddings that encode latent biological associations. Second, query-guided subgraph construction and propagation (Section 2.2.3) iteratively builds a query-relevant subgraph by refining relations and propagating context-specific embeddings. Finally, these complementary aspects are unified through a hybrid scoring mechanism (Section 2.2.4). This mechanism integrates KE’s direct global semantic contributions with structural insights from the KE-informed GSP process, enabling a nuanced assessment of candidate predictions.

#### 2.2.1 Notations and problem setup

Let G=(V,R,F,Q) be a biomedical KG integrating diverse fact triples from multiple sources for various tasks, as shown in Fig. 1A. Here, V is the set of entities and R the set of relations. F is the set of factual triples, F={(h,r,t)∣h,t∈V,r∈R}, where head entity *h* and tail entity *t* are connected by relation *r*. To enhance graph diversity and model robustness, we also incorporate triples with reverse and identity relations (Zhang and Yao 2022, Zhang *et al.* 2023). Q contains query triples, $Q=\{(q_{e},q_{r},q_{a})|q_{e},q_{a}\in V,q_{r}\in R\}$. Each query is of the form $\left(q_{e},q_{r},?\right)$, with $q_{a}$ as the unknown target entity. The objective for such queries is to predict $q_{a}$, a task central to KGC and KGR aimed at enriching the KG.

#### 2.2.2 Tensor decomposition and query initialization

Effective biomedical knowledge analysis hinges on understanding the global semantic landscape to foster a dynamic interplay between semantic insights and structural patterns. BioGraphFusion initiates this by establishing a global semantic foundation through tensor decomposition of the entire KG. This initial step provides a rich context essential for the subsequent integration of structural patterns with semantic understanding. For this critical stage, we employ CP decomposition (Kolda and Bader 2009). CP is chosen as it directly factorizes the graph’s adjacency tensor to derive meaningful, low-dimensional latent embeddings for entities and relations. This factorization process adeptly captures fundamental relationships. Moreover, CP’s formulation as a low-rank tensor approximation offers a balance between model expressiveness and parsimony, ensuring computational efficiency and scalability vital for processing large-scale biomedical KGs.

The graph tensor $T\in R^{\left|V|\times |R|\times |V\right|}$ is factorized via CP into three matrices: $E_{h}\in R^{|V|\times D}$, $E_{r}\in R^{|R|\times D}$, and $E_{t}\in R^{|V|\times D}$. These matrices capture the latent semantic associations between entities and relations (Fig. 1B1). The compatibility of any triple (h,r,t) is then computed as:

(1) $$\phi (h,r,t)=\sum_{d=1}^{D}e_{h}^{\left(d\right)}\cdot e_{r}^{\left(d\right)}\cdot e_{t}^{\left(d\right)},$$

where $e_{h}^{\left(d\right)}$, $e_{r}^{\left(d\right)}$, and $e_{t}^{\left(d\right)}$ are the *d*th components of the respective embeddings.

Subsequently, BioGraphFusion initializes query-specific representations directly from the CP-extracted matrices, ensuring a semantically meaningful starting point. Specifically, given a query $\left(q_{e},q_{r},?\right)$, the entity embedding $e_{q_{e}}$ and the initial relation embedding $e_{q_{r}}^{0}$ are retrieved from $E_{h}$ and $E_{r}$, respectively (Fig. 1B2). The initial node representation $h^{0}$ is thus set to $e_{q_{e}}$, establishing a query-grounded context before neighborhood expansion. Similarly, all entity and relation embeddings in the graph, including those encountered during propagation, are initialized from CP decomposition, preserving global structural information for subgraph construction and message passing.

**Figure 2.**
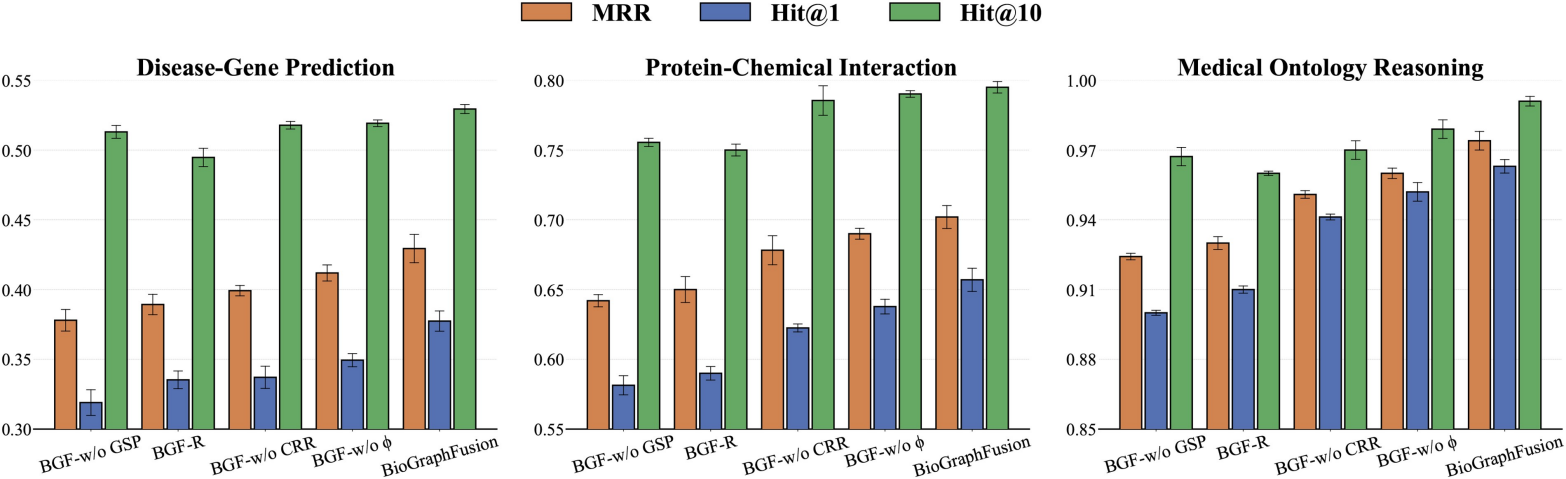
Ablation study results for BioGraphFusion (BGF) across three biomedical reasoning tasks: disease-gene prediction, protein-chemical interaction, and medical ontology reasoning. Performance metrics include MRR, Hit@1, and Hit@10. The full model is compared against four ablated variants: BGF-w/o GSP, BGF-R, BGF-w/o CRR, and BGF-w/o ϕ.

#### 2.2.3 Query-guided subgraph construction

Biomedical KGs are vast and noisy, making it computationally impractical and error-prone to process the entire graph for each query. To address this, we employ a query-guided subgraph construction mechanism that selectively expands along semantically relevant paths (see Fig. 1C), ensuring biological meaningfulness while filtering out spurious connections.

**Neighborhood expansion** At each layer ℓ, the model expands the neighborhood for further propagation. Initially, at ℓ=0, the entity set $V^{\left(0\right)}$ contains only the query node $q_{e}$. For each entity *h* at layer ℓ−1, we construct the candidate set $C^{\left(\ell \right)}$ by aggregating all direct neighbors of the current nodes:

(2) $$C^{\left(\ell \right)}=\underset{h\in V^{\left(\ell -1\right)}}{\cup }\{t|(h,r,t)\in F\}.$$

In this step, the model gathers all possible entities that can serve as neighbors for the current nodes during propagation. On that basis, standard GNNs often update each node representation iteratively by gathering information from the surrounding entities. We also follow this step in our approach to constructing a candidate set $C^{\left(\ell \right)}$ to prepare for message propagation.

**Contextual relation refinement** In many existing approaches, relation embeddings remain static or minimally updated, failing to account for contextual variations. However, in biomedical KGs, relations are rarely fixed; their meaning is shaped by the entities involved and the reasoning path. For instance, the relation “disease_gene” can imply different biological mechanisms depending on the specific genes or proteins connected. Furthermore, static embeddings struggle to model multi-step interactions, such as indirect associations mediated by proteins or chemicals.

To mitigate these limitations, we introduce a contextual relation refinement (CRR) module. LSTMs are chosen for their stateful transformation and gating mechanisms, which allow them to effectively model how relation meanings vary with entity context—a common scenario in biomedical KGs. Unlike simpler recurrent units (e.g., RNNs, GRUs), LSTMs excel at refining relation representations based on evolving semantic contexts from connected entities. This yields context-specific embeddings better suited for the dynamic, multi-step nature of biomedical relationships, iteratively updating relation embeddings as well as capturing context-dependent semantics and long-range dependencies (Wang *et al.* 2024a). Specifically, for each triple (h,r,t), the LSTM updates the relation embedding $e_{r}^{\ell }$ at layer ℓ, using the previous embedding $e_{r}^{\ell -1}$ as input and the head entity embedding $e_{h}$ as the hidden state:

(3) $$e_{r}^{\ell }=\text{LSTM}\;(e_{r}^{\ell -1},e_{h}).$$

Through the internal gating mechanisms (including the forget gate *f*, input gate *i*, candidate memory cell $\tilde{c}$, memory cell *c*, and output gate *o*), LSTM selectively processes and retains relevant contextual information. It tailors the relation embedding to the connected entities. Similarly, the query relation $e_{q_{r}}^{\ell }$ is updated by:

(4) $$e_{q_{r}}^{\ell }=\text{LSTM}\;(e_{q_{r}}^{\ell -1},e_{q_{e}}).$$

The dual LSTM adaptively refines both head-node and query relation representations based on semantic context from their respective entity embeddings. This dynamic modulation helps the model grasp nuanced relationships. Comparative experiments (see Section 8, available as supplementary data at *Bioinformatics* online for details) have confirmed that LSTMs are better than other alternatives, validating the capability of achieving deep semantic-structural coupling central to our model.

**Query-attention propagation** Inspired by RED-GNN (Zhang and Yao 2022), each candidate node *t* aggregates messages from its neighbors using a query-attentive mechanism (Fig. 1C2). Specifically, the node representation at layer ℓ is updated as

$$h_{t}^{\ell }(q_{e},q_{r})=\delta \left(W^{\ell }\cdot \sum_{(h,r,t)\in C^{\left(\ell \right)}}\alpha_{h,r,t|q_{r}}^{\ell }\left(h_{h}^{\ell -1}(q_{e},q_{r})+e_{r}^{\ell }\right)\right),$$

where $W^{\ell }$ is a trainable weight matrix and δ denotes the Tanh activation function. The attention weight $\alpha_{h,r,t|q_{r}}^{\ell }$, computed as

$$\alpha_{h,r,t|q_{r}}^{\ell }=\sigma \left({\left(w_{\alpha }^{\ell }\right)}^{⊤}\text{ReLU}\left(W_{\alpha }^{\ell }\cdot \left[h_{h}^{\ell -1}(q_{e},q_{r})+e_{r}^{\ell }+e_{q_{r}}^{\ell }\right]\right)\right)$$

integrates both local neighborhood features and the global query context, with $e_{q_{r}}^{\ell }$ being the query-specific relation embedding refined by the LSTM module.

**Biological relevance filtering** Following AdaProp (Zhang *et al.* 2023), after node representations are updated, we compute an importance score for each candidate node *t*:

(5) $$s_{t}=W_{\text{samp}}\cdot h_{t}^{\ell }(q_{e},q_{r}).$$

This score quantifies the biological relevance of each node. We then filter the candidate set by retaining only the top *K* nodes. During training, the top *K* nodes are selected via a differentiable Gumbel-Softmax, while during inference, a conventional Softmax selection is applied:

(6) $$V^{\left(\ell \right)}=\text{TopK}\left(s_{t}|t\in C^{\left(\ell \right)}\right).$$

For details on gradient-preserved hard selection, see Section 2, available as supplementary data at *Bioinformatics* online.

**Final subgraph construction** Building upon this iteration, the final subgraph $G_{q}$ is constructed over ℓ layers (Fig. 1D):

(7) $$G_{q}=(V_{q},E_{q}),$$

where $V_{q}$ denotes the set of selected entities and $E_{q}$ the relationships among them. This refined subgraph, enriched with contextually relevant information, is then used for downstream tasks such as KG completion and reasoning, ensuring that only the most pertinent interactions are propagated.

#### 2.2.4 Joint formulation of scoring and loss functions

Focusing only on graph message or knowledge representation in the final scoring function may miss complementarity. Pure graph modeling may overlook deeper semantic relationships, whereas embedding methods might not capture fine-grained structural details. To better leverage the advantages of both perspectives, BioGraphFusion incorporates elements from KE and graph propagation into its final scoring function. For a triple $\left(q_{e},q_{r},q_{a}\right)$, our score is defined as a weighted sum (see Fig. 1E):

(8) $$\tilde{f}(q_{e},q_{r},q_{a})=\lambda f(q_{e},q_{r},q_{a})+(1-\lambda )\phi (q_{e},q_{r},q_{a}),$$

where λ∈[0,1] balances the contributions from two key components. f(·) represents the score derived from the KE-informed graph propagation process, capturing contextualized structural patterns, whereas ϕ(·) provides a direct global semantic score obtained through tensor decomposition. The hybrid design combines semantic knowledge with graph propagation through bidirectional interactions to refine structural representations. The component $f(q_{e},q_{r},q_{a})$ is computed from the final representation of the target entity obtained via iterative message passing:

(9) $$f(q_{e},q_{r},q_{a})=w^{⊤}h_{q_{a}}^{\ell }(q_{e},q_{r})$$

and the tensor decomposition–based score, capturing the global biological context, is given by

(10) $$\phi (q_{e},q_{r},q_{a})=\sum_{d=1}^{D}e_{q_{e}}^{\left(d\right)}\cdot e_{q_{r}}^{\left(d,\ell \right)}\cdot e_{q_{a}}^{\left(d\right)}$$

with $e_{q_{e}}^{\left(d\right)}$, $e_{q_{a}}^{\left(d\right)}$, and $e_{q_{r}}^{\left(d,\ell \right)}$ denoting the *d*th components of the CP embeddings for the query entity, target entity, and the refined query relation (updated at layer ℓ using an LSTM that incorporates $e_{q_{e}}$), respectively.

To train BioGraphFusion for biomedical completion and reasoning, we design a composite loss function with two objectives: (i) to maximize the likelihood of true relationships and (ii) to learn robust, generalizable embeddings. The primary component is a multi-class log-loss that encourages the model to assign higher scores to positive triples from the training set $F_{\text{tra}}$ compared to negative candidates. Specifically, the log-loss is defined as:

$$L_{\;\text{log}\;}=\sum_{(q_{e},q_{r},q_{a})\in F_{\text{tra}}}\left[-\tilde{f}(q_{e},q_{r},q_{a})+\text{log}\;\sum_{t\in V}\;\text{exp}\;\left(\tilde{f}(q_{e},q_{r},t)\right)\right].$$

In addition, following CP-N3 (Lacroix *et al.* 2018), we incorporate an N3 regularization term. The primary motivation for this is to penalize large magnitudes in CP embeddings, thereby mitigating overfitting:

(11) $$R_{N3}=|eq_{e}{|}_{3}^{3}+|eq_{r}{}^{\ell }{|}_{3}^{3}+|e_{q_{a}}{|}_{3}^{3}.$$

To further validate our choice of N3, ablation studies on regularization were conducted (see Section 8, available as supplementary data at *Bioinformatics* online). These studies have confirmed the robustness of our model architecture, demonstrating that the model performs well and outperforms baselines even when employing naive regularizations (L1 or L2). Notably, the N3 regularization, generally yields superior results over alternatives. This advantage is attributed to the selection of the optimized configuration, reinforcing its suitability for our approach.

Thus, the overall loss is given by:

(12) $$L=L_{\;\text{log}\;}+\gamma R_{N3},$$

where γ controls the regularization strength.

## 3 Results

### 3.1 Implementation details

**Experimental setup.** All experiments were implemented in Python using PyTorch v1.12.1 and PyTorch Geometric v2.0.9 on a single NVIDIA RTX 3090 GPU. Key hyperparameters were tuned over specific ranges; detailed configurations are provided in Section 3, available as supplementary data at *Bioinformatics* online.

**Evaluation metrics and baseline competitors.** Following Zhang *et al.* (2023) and Zhang and Yao (2022), we evaluate model performance using filtered ranking-based metrics: mean reciprocal rank and Hit@*k* (with k=1 and 10). Detailed definitions of these metrics are provided in Section 4, available as supplementary data at *Bioinformatics* online. We benchmark BioGraphFusion against state-of-the-art methods from three major categories: KE models, GSP (GNN-based) approaches, and Ensemble methods. All baselines are implemented using publicly available code from the respective authors. Comprehensive descriptions of these baselines and implementation details are provided in Section 5, available as supplementary data at *Bioinformatics* online.

**Datasets and data integration.** The disease–gene Prediction task uses 130 820 disease–gene associations from DisGeNET (Piñero *et al.* 2020), partitioned 7:2:1 (training:validation:test) based on a specific fold from the KDGene (Wang *et al.* 2024a) 10-fold cross-validation setup. For a comprehensive generalization assessment, we also conduct full 10-fold cross-validation (see Section 6, available as supplementary data at *Bioinformatics* online). Supplementary data, available as supplementary data at *Bioinformatics* online include 14 631 drug–disease relationships from SIDER (Kuhn *et al.* 2016) and 277 745 protein-chemical interactions from STITCH (Szklarczyk *et al.* 2016). The Protein–Chemical Interaction task uses 23 074 interaction triples from STITCH, filtered to the top 100 most frequent genes (Wang *et al.* 2024b), and partitioned 7:2:1. To address data imbalance from extensive background knowledge, we cap supplementary samples at 15 000 for disease–gene association prediction and 10 000 for protein–chemical interaction. The medical ontology reasoning task is based on the UMLS Terminology (Bodenreider 2004), pre-split into background, training, validation, and test sets as in prior work (Zhang and Yao 2022, Zhang *et al.* 2023). Further dataset and task details are in Section 1, available as supplementary data at *Bioinformatics* online.

### 3.2 Overall performance

Table 1 shows BioGraphFusion consistently outperforms KE, GNN, and Ensemble baselines across all three tasks. Regarding computational efficiency, Section 7, available as supplementary data at *Bioinformatics* online compares the inference time and MRR performance of BioGraphFusion with competitive baseline models on the UMLS dataset, analyzing the trade-off between their predictive performance and computational efficiency.

**Table 1.** Evaluation results of BioGraphFusion on biomedical completion and reasoning.^a^

Type	Models	Disease–gene prediction			Protein–chemical interaction			Medical ontology reasoning		
		MRR	Hit@1	Hit@10	MRR	Hit@1	Hit@10	MRR	Hit@1	Hit@10
**KE**	RotatE	0.263	0.202	0.381	0.606	0.512	0.778	0.925	0.863	0.993
	ComplEx	0.392	0.336	0.498	0.356	0.236	0.594	0.630	0.493	0.893
	DistMult	0.258	0.198	0.375	0.120	0.045	0.276	0.569	0.461	0.797
	CP-N3	0.207	0.151	0.312	0.089	0.029	0.189	0.300	0.134	0.750
	KDGene	0.384	0.321	0.523	0.085	0.023	0.170	0.260	0.100	0.708
**GNN**	pLogicNet	0.228	0.173	0.335	0.591	0.564	0.630	0.842	0.772	0.965
	CompGCN	0.252	0.191	0.367	0.614	0.576	0.676	0.907	0.867	0.994
	DPMPN	0.293	0.235	0.393	0.632	0.614	0.729	0.930	0.899	0.980
	AdaProp	0.345	0.296	0.438	0.662	0.631	0.781	0.969	0.956	**0.995**
	RED-GNN	0.389	0.332	0.468	0.662	0.613	0.782	0.964	0.946	0.990
**Ensemble**	KG-BERT	–	–	–	–	–	–	0.774	0.649	0.967
	StAR	0.247	0.192	0.361	0.426	0.326	0.700	0.834	0.720	0.976
	LASS	0.211	0.167	0.324	0.401	0.314	0.691	0.908	0.952	0.983
	Ours	0.429**	0.377**	0.529*	0.702**	0.657*	0.795*	0.974	0.963*	0.991

a “-” means unavailable results. The best results are highlighted in bold and the second-best results are underlined.

* denotes statistically improvements over the best baseline (*P < .01, **P < .001, paired *t*-test on five random seeds).

**KE methods** reveal limitations in pure embedding approaches. ComplEx achieves moderate success in disease–gene association prediction (MRR 0.392) by modeling asymmetric relations, while RotatE (MRR 0.263) struggles despite its sophisticated rotation-based relation modeling. CP-N3’s poor performance in protein–chemical interaction is more telling. While CP-N3 uses tensor decomposition, a principle foundational to our approach, its standalone application, lacking crucial integration with structural learning, highlights the limitations of relying solely on this embedding technique. Even KDGene, engineered for disease-gene associations using interactional tensor decomposition, achieves only 0.384 MRR, showing semantic modeling alone, without adaptive structural guidance, cannot fully capture intricate biomedical dependencies.

**GNN approaches** show different strengths and limitations. RED-GNN performs strongly in Disease-Gene Prediction (MRR 0.389), and AdaProp excels in Protein-Chemical Interaction (MRR 0.662). However, their weakness of reliance on structural patterns is apparent when compared to BioGraphFusion, which demonstrates consistent improvements in these tasks. While AdaProp has a slight edge in highly structured UMLS tasks, the merit diminishes in more semantically complex biomedical scenarios. While effective for local connectivity, pure structural propagation lacks the global semantic context needed to interpret biological relationships.

**Ensemble methods** exhibit limitations in biomedical contexts. KG-BERT performs moderately in medical ontology reasoning (MRR 0.774), while StAR and LASS show limited effectiveness in Disease-Gene Association Prediction. A key constraint is their textual encoding components’ limitation by sparse entity information—biomedical entities are often identifiers or technical terms, not descriptive text. This yields shallow semantic embeddings, hindering effective structural integration. While these methods try to bridge semantic understanding with structural patterns (StAR via Siamese encoding, LASS via joint fine-tuning), their limitations show that effective biomedical ensemble integration needs more than combining components.

**BioGraphFusion’s superior performance** stems from its innovative deep coupling between semantic understanding and structural learning. Unlike existing methods that combine these paradigms statically, our model enables dynamic co-evolution where semantic insights guide structural reasoning while structural discoveries enrich semantic understanding. This deep coupling effectively models the intricate, context-dependent relationships in biomedical KGs, resulting in significant performance improvements across diverse tasks.

### 3.3 Ablation study

To evaluate individual component contributions in BioGraphFusion, we performed ablation studies on key modules for global semantics, GSP, and their hybrid scoring. Four targeted variants were implemented: (i) removal of GSP (BGF-w/o GSP): removes dynamic structural learning to assess GSP’s role in our model; (ii) random query encoding (BGF-R), in which the CP-derived query embeddings are replaced with randomly initialized vectors, disrupting semantic alignment; (iii) removal of contextual relation refinement (BGF-w/o CRR), which omits the LSTM-based updates for relation embeddings; and (iv) Elimination of the Tensor Decomposition Score (BGF-w/o ϕ), which excludes the CP-based branch from the hybrid scoring function, leaving only the contextualized structural patterns to drive the scoring mechanism.

#### 3.3.1 Performance comparison

Figure 2 summarizes the ablation study results, highlighting the distinct contributions of structural propagation (GSP) and KE components. Removing the GSP module (BGF-w/o GSP) leads to the most pronounced performance drop across all tasks, underscoring the essential role of dynamic structural learning in capturing topological dependencies and facilitating effective knowledge integration. This result demonstrates that structural propagation is indispensable for modeling complex biomedical relationships that rely on multi-hop and context-dependent interactions.

In contrast, the other three ablation variants—random query encoding (BGF-R), removal of contextual relation refinement (BGF-w/o CRR), and elimination of the tensor decomposition score (BGF-w/o ϕ)—primarily target KE-related modules. Each of these modifications results in significant but distinct performance declines. BGF-R confirms the necessity of CP-based semantic initialization for maintaining meaningful entity representations; BGF-w/o CRR highlights the importance of LSTM-driven contextual refinement for relation embeddings; and BGF-w/o ϕ demonstrates that optimal performance requires balancing global semantic signals with graph-derived structural patterns. Collectively, these findings confirm that our model’s success stems from the synergy between structural propagation and semantic embedding, not from either component alone.

#### 3.3.2 Semantic embedding visualization

**Figure 3.**
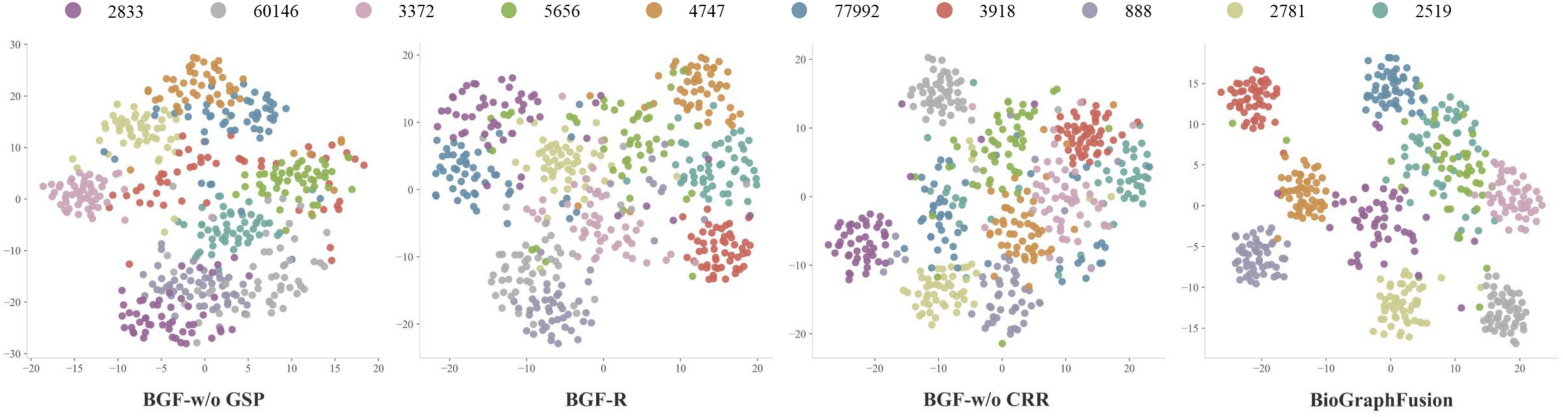
t-SNE visualization of protein embeddings. Each subfigure shares the same proteins and each color represents proteins interacting with the same chemical compound, labeled by PubChem CID.

To assess KE components’ impact on semantic representation, we visualize protein embeddings from the protein–chemical interaction task using t-SNE (Fig. 3). We selected 10 chemical compounds (each linked to 50–100 proteins) and compared the full BioGraphFusion model with ablation variants BGF-w/o GSP, BGF-R, and BGF-w/o CRR. The BGF-w/o ϕ variant was excluded as its scoring function primarily affects prediction scores, not embedding coordinates. Protein embeddings were obtained via post-propagation representations for GSP variants (full, BGF-R, BGF-w/o CRR), and via final CP embeddings for BGF-w/o GSP.

The t-SNE visualizations in Fig. 3 illustrate progressive improvement in semantic coherence as key architectural components are integrated. The full BioGraphFusion model produces optimally tight and well-separated protein embeddings for each chemical compound, showing strong intra-cluster cohesion and inter-cluster separation. Conversely, BGF-w/o GSP (relying solely on initial CP embeddings) shows the most diffuse clustering with indistinct inter-group boundaries, highlighting GSP’s role in refining entity distinctions. BGF-R (with random query embeddings) exhibits clustering with significant overlap, confirming that effective GSP depends on high-quality initial semantic representations. BGF-w/o CRR shows clearer clustering than the previous two variants (benefiting from CP initialization and GSP), yet its clusters are less separated than the full model, emphasizing the crucial role of LSTM-driven relation refinement in forming clear, coherent clusters. These results confirm that CP initialization, dynamic GSP, and LSTM relation refinement each make unique contributions to meaningful biomedical entity representations. Visualization results for competitive baselines in Section 9, available as supplementary data at *Bioinformatics* online generally show more diffuse embedding clusters, further demonstrating BioGraphFusion’s effectiveness.

### 3.4 Hyperparameter sensitivity analysis

We conducted extensive hyperparameter tuning on the disease–gene prediction task to examine the impact of key parameters on the final performance of BioGraphFusion. In our experiments, we varied the batch size, embedding dimension *D*, fusion weight λ, and the number of propagation steps ℓ. Our results on the disease–gene dataset indicate optimal performance with a batch size of 16, an embedding dimension D=32, a fusion weight λ=0.7, and ℓ=6 propagation steps. Notably, the model enjoys robustness to batch size variations, while an embedding dimension of D=32 is found to effectively capture semantic details without over-parameterization. Tuning λ and ℓ reveals critical balances: λ=0.7 optimally harmonizes structural propagation with global semantic embeddings, while ℓ=6 propagation step effectively balances information aggregation against over-smoothing. This careful hyperparameter calibration is vital for maximizing model performance on biomedical tasks. Further details are in Section 10, available as supplementary data at *Bioinformatics* online.

### 3.5 Case analysis of cutaneous malignant melanoma 1

#### 3.5.1 Pathogenic gene prediction

We used BioGraphFusion to predict ten candidate genes for cutaneous malignant melanoma 1 (CMM1), including two known disease-associated genes (CDKN2D and CDK4) and eight novel candidates (Table 2). To validate these predictions (Pred.), we cross-referenced the candidates against three independent databases: PubMed (using PMIDs), MalaCards and ClinVar. We found that seven of the eight novel candidate genes—AKT1 (rank 3), NF1 (rank 5), OCA2 (rank 6), TP53 (rank 7), TYRP1 (rank 8), TYR (rank 9), and NRAS (rank 10)—are documented in both MalaCards and ClinVar, indicating established associations with melanoma or related conditions. Additionally, PubMed searches revealed literature support for the co-occurrence of CMM1 with all eight novel candidates.

**Table 2.** For CMM1, top 10 candidate gene predicted by BioGraphFusion.

Rank	Pred.	PMIDs	MalaCards	ClinVar
1	CDKN2D^a^	–	–	–
2	CDK4D^a^	–	–	–
3	AKT1	38275910,39659584	✓	✓
4	HPS1	15982315,23084991		
5	NF1	38179395,37965626	✓	✓
6	OCA2	37646013,37568588	✓	✓
7	TP53	24919155,38667459	✓	✓
8	TYRP1	37646013,37239381	✓	✓
9	TYR	19578364,18563784	✓	✓
10	NRAS	38275910,38183141	✓	✓

a These genes predicted by BioGraphFusion are in the test set.

#### 3.5.2 Pathway enrichment and protein–protein interaction analysis

**Figure 4.**
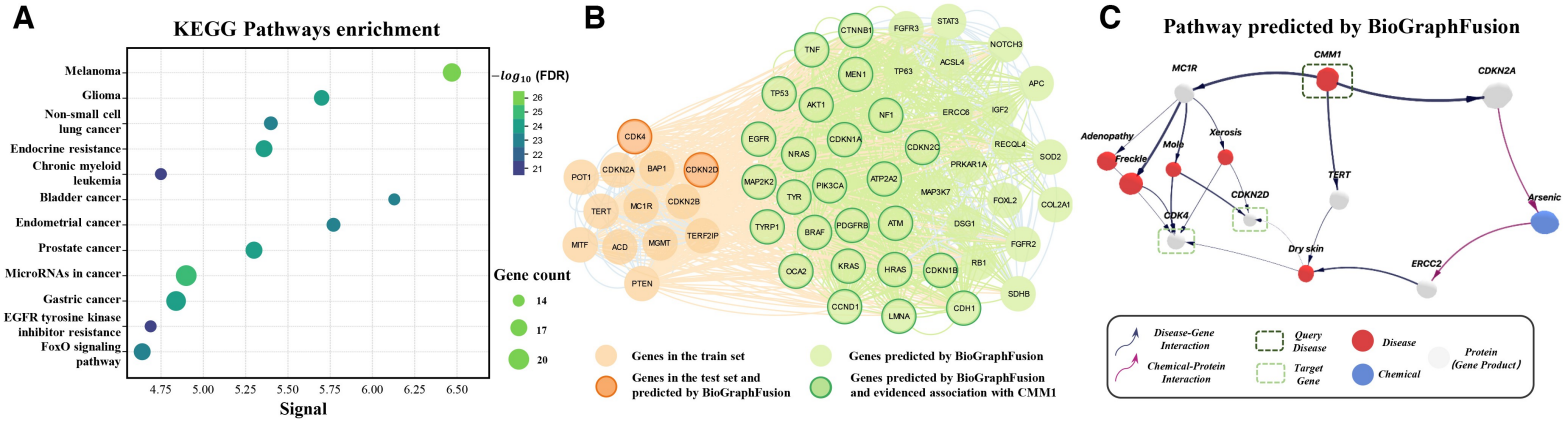
Case study. (A) Analysis of KEGG pathway enrichment for the benchmark. The bubble chart shows significantly enriched pathways related to melanoma pathways. (B) Link visualization of known and predicted genes for melanoma on the PPI network. (C) Pathway predicted by BioGraphFusion from query disease CMM1 to melanoma-associated genes reveals a biologically plausible mechanistic link between CMM1 and established melanoma genes.

To evaluate the biological relevance of both known genes and the predictions for CMM1, we performed a KEGG pathway enrichment analysis. Figure 4A presents the top 12 enriched pathways; notably, the “Melanoma” pathway shows the strongest enrichment (FDR = 2.20e−26) with 18 prominently represented genes. In addition, pathways associated with Glioma and non-small cell lung cancer were also enriched, further supporting the biological plausibility of the candidate genes.

We further employed CMM1 as an illustrative example to evaluate the network proximity and functional coherence between genes in the train set and the candidate genes predicted by BioGraphFusion. For this analysis, we retain all 11 genes from the training set and 2 genes from the testing set of the DisGeNET dataset, and extract the top 50 candidate genes predicted by BioGraphFusion. The resulting protein–protein interaction network (Fig. 4B) exhibits markedly denser connectivity than would be expected by chance (*P* = 4.669E−86, binomial test). Detailed connectivity statistics and analysis are provided in Section 11, available as supplementary data at *Bioinformatics* online. This dense interconnectivity suggests that the candidates are functionally related to the known genes, reinforcing the biological relevance of our predictions.

#### 3.5.3 Pathway reasoning and biological validation

By analyzing inference pathways that connect candidate genes to known disease ones, we aim to infer their functional relationships to discover the causative mechanism. For example, analyzing pathways linking disease CMM1 to known melanoma-associated genes CDKN2D and CDK4 (Fig. 4C), with edge thickness representing attention weights, revealed a key pathway (CMM1 → MC1R → Mole → CDK4/CDKN2D) offering novel insights into melanoma pathogenesis.

To further understand the mechanisms our model holds, we examined the inferred CMM1 → MC1R → Mole pathway, strongly backed by existing biological evidence. Research by Su *et al.* (2023) shows a progressive increase in MC1R expression throughout melanoma development, from benign moles to metastatic melanoma. Separately van der Poel *et al.* (2020) identified the MC1R *Val60Leu* variant as a significant predictor for high mole counts, confirming the MC1R-Mole link. Together, these findings support this pathway’s biological plausibility, suggesting a coherent mechanism in melanoma pathogenesis.

Notably, an alternative pathway, CMM1 → MC1R → Freckle, also receives high attention weights. This aligns with (Bastiaens *et al.* 2001), who linked MC1R variants to freckle formation, reinforcing its connection to CMM1. As illustrated in Fig. 4C, other MC1R-associated conditions, many with dermatological manifestations, show varying correlations with melanoma. These identified pathways deepen our understanding of disease mechanisms and highlight potential research directions.

## 4 Discussion

Building on the demonstrated success of BioGraphFusion, particularly in the CMM1 case study, our future work will focus on two key areas. We plan to validate the framework's efficacy across a broader spectrum of complex diseases to test its generalizability. Concurrently, we aim to enhance our model's core synergistic mechanism to integrate multi-modal data, such as clinical texts, further deepening the interplay between semantic and structural learning for more comprehensive biomedical discovery.

## 5 Conclusion

In this work, we introduce BioGraphFusion, a novel framework synergistically integrating semantic understanding with structural learning for biomedical KGC and KGR. BioGraphFusion enhances the dynamic interplay between these paradigms by using CP decomposition to establish a global semantic context. Building upon this, an LSTM-driven mechanism guides structural learning by dynamically refining relational information and updating semantic understanding during graph propagation. This enables learning context-dependent relation semantics and captures long-range dependencies, moving beyond static interpretations. Complemented by query-guided subgraph construction and a hybrid scoring mechanism, BioGraphFusion fosters a deep, adaptive refinement cycle between structural learning and semantic comprehension. Experimental results show BioGraphFusion consistently outperforms traditional KE models, GNN-based approaches, and ensemble methods across biomedical benchmarks. Its ability to generate comprehensive features through an effective synergy of semantic insights and structural learning establishes it as a powerful tool. Finally, as demonstrated in the CMM1 case study, its capacity to uncover biologically meaningful pathways highlights its potential for advancing biomedical research.

## Supplementary Material

btaf408_Supplementary_Data
