# Supplementary material for: BioGraphFusion: graph knowledge embedding for biological completion and reasoning
Source: Bioinformatics. 2025 Jul 18;41(7):btaf408. doi: 10.1093/bioinformatics/btaf408 (PMC12311287; doi:10.1093/bioinformatics/btaf408)
Supplement: btaf408_Supplementary_Data [file btaf408_supplementary_data.pdf]

Supplementary Materials for BioGraphFusion: Graph Knowledge Embedding for Biological Completion and Reasoning

Overall of the BioGraphFusion

BioGraphFusion is a novel framework meticulously engineered to transcend the limitations of prior methods by enabling a truly synergistic integration of semantic understanding and structural learning within biomedical knowledge graphs (KGs). Its architecture, illustrated in Fig. 1, is designed to foster deep interaction mechanisms, ensure dynamic structural learning under global semantic guidance, and achieve adaptive refinement through the coupled interplay of its components, thereby fostering a profound and dynamic coupling between these two paradigms.

The process commences with the construction of a biomedical KG from factual triples relevant to the specific task. For instance, Disease-Gene Prediction tasks might utilize graphs built from Drug-Disease and Protein-Chemical relationships, while Medical Ontology Reasoning would leverage diverse medical relationships to ensure domain-specific fidelity.

At the heart of BioGraphFusion's strategy to overcome the lack of overarching semantic direction in many previous models is its Global Biological Tensor Encoding module. This component employs Canonical Polyadic (CP) decomposition to generate initial, rich embedding matrices for head entities, relations, and tail entities. Crucially, these embeddings establish a foundational global semantic context, capturing latent biological associations and providing the essential top-down guidance for the subsequent, more nuanced and dynamic structural exploration that follows.

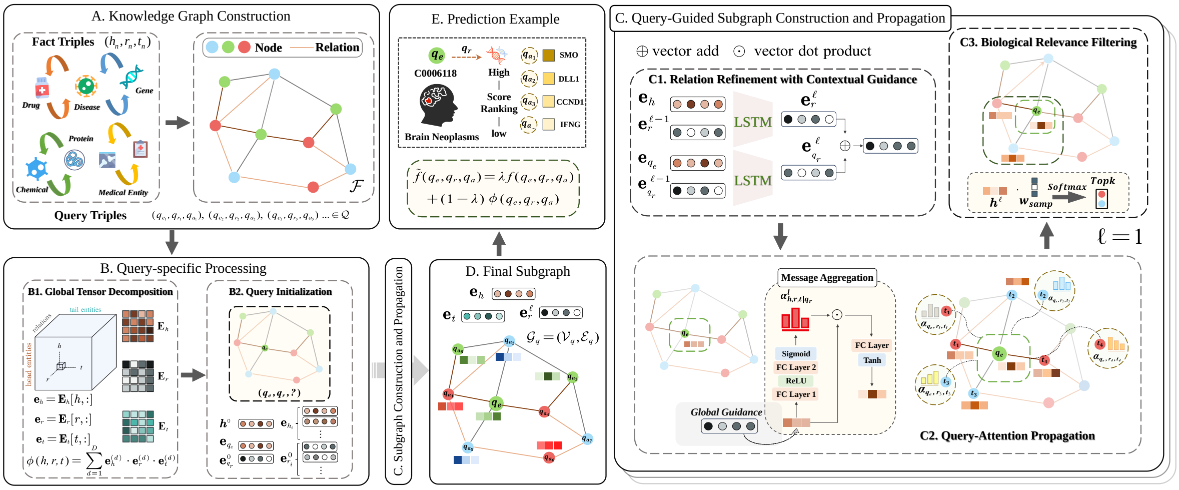

Fig. 1: Overview of the BioGraphFusion framework. (A) Knowledge Graph Construction: Integrating biomedical datasets to form a unified knowledge graph for downstream tasks. (B) Query-Specific Processing: A two-step process involving (B1) Global Tensor Decomposition that captures latent biological associations, and (B2) Query Initialization that guides the search process. (C) Subgraph Construction and Propagation, including (C1) Relation Refinement via LSTM, (C2) Query-Attention Propagation with context-based attention weights, and (C3) Biological Relevance Filtering to select the most pertinent entities. (D) Final Subgraph. (E) Scoring Integration that balances structural and semantic information and Prediction Example that selects the most promising predictions, with a focus on query disease Brain Neoplasms.

Building upon this global semantic context, the Query-Guided Subgraph Construction and Propagation mechanism is introduced, playing a pivotal role in realizing BioGraphFusion's dynamic and adaptive capabilities. This module first iteratively constructs a query-relevant subgraph by selectively expanding along paths deemed semantically meaningful,

© The Author 2025. Published by Oxford University Press. All rights reserved. For permissions, please e-mail: journals.permissions@oup.com

guided by the global embeddings and the evolving query context. A core innovation within this propagation process is the LSTM-driven Contextual Relation Refinement. This powerful mechanism dynamically refines relation embeddings during multi-layer propagation within the focused subgraph. Such dynamic refinement allows these embeddings to adapt to evolving semantic contexts and capture long-range dependencies—a clear departure from static representations. Critically, this LSTM-driven process ensures that structural learning is continuously informed by and interacts with semantic insights, fostering a profound and dynamic coupling between these two paradigms. Complementing this deep, contextual refinement of relations, the query-guided nature of the subgraph construction further focuses structural exploration and message passing on the most biologically pertinent regions, enabling highly adaptive refinement and targeted learning.

Finally, to ensure a holistic and robust prediction, A hybrid scoring mechanism synthesizes the knowledge gained. This mechanism is aimed at achieving an optimal balance between the foundational global semantic knowledge (captured by the initial tensor embeddings) and the contextualized structural representations derived from the dynamic, semantically-guided graph propagation. This step ensures that both the broad, overarching associations and the fine-grained, dynamically uncovered structural patterns contribute to the final output.

In essence, by systematically establishing global semantic guidance, enabling dynamic and context-aware structural learning that is continuously informed by semantic insights, and ensuring their deep, adaptive coupling, BioGraphFusion's architecture is designed to deliver more accurate predictions and profound biological insights, moving beyond the capabilities of models with less integrated semantic and structural processing.

(1) SM1: Experimental Tasks and Datasets

All experiments were conducted using the datasets presented in Table 1. The table details three main tasks: prediction of disease genes, protein-chemical interactions, and medical ontology reasoning, each accompanied by specific background knowledge. Together, these datasets offer a comprehensive and robust foundation for evaluating our methods across multiple biomedical domains.

Table 1. Datasets and Task Overview.

**Tasks**	**Background Knowledge Sources**	**Main Datasets Targets**
Disease-Gene Prediction	Drug-Disease Relationships<br>SIDER (14,631)<br>Protein-Chemical Relationships<br>STITCH (277,745)	DisGeNet (130,820)<br>Gene
Protein-Chemical Interaction	Drug-Disease Relationships<br>SIDER (14,631)<br>Disease-Gene Relationships<br>DisGeNet (130,820)	STITCH (23,074)<br>Chemical
Medical Ontology Reasoning	Various Medical Relationships<br>UMLS(4,006)	UMLS(2,523)<br>Multi-domain Entities

Disease-Gene Prediction task: This task aims to identify gene entities associated with a given disease; for example, if the input is "Alzheimer's Disease", the model would predict associated genes such as "APOE". We utilize 130,820 disease-gene associations from the DisGeNET database, following the data foundation of the KDGene study. To provide broader contextual information, this primary dataset is enriched by incorporating drug–disease relationships from SIDER (Kuhn et al., 2016) and protein–chemical interactions from STITCH (Szklarczyk et al., 2016). While these sources offer a substantial number of potential contextual interactions (14,631 and 277,745 respectively), to mitigate potential data imbalance from such extensive background knowledge and maintain a focused set of supplementary data, the number of supplementary samples utilized from these enrichment sources for the Disease-Gene Prediction task was capped at 15,000. Our primary experiments, detailed in this manuscript, were based on one specific fold selected from KDGene's 10-fold cross-validation setup (which uses a 90% training/10% testing split per fold). Within this chosen fold, KDGene's original 10% segment served as our test set. The remaining 90% (KDGene's training portion) was partitioned by us into a 70% training set and a 20% validation set for BioGraphFusion, relative to the total data in that fold. This established an effective 70%/20%/10% train/validation/test configuration, where the validation set was used for hyperparameter optimization.

To provide a more comprehensive assessment of BioGraphFusion's generalization performance and robustness, and for direct comparison under KDGene's full benchmark protocol, we also conducted a complete 10-fold cross-validation. In this broader evaluation, for each of the 10 original KDGene folds, BioGraphFusion was trained directly on the entire 90% training data portion defined by KDGene for that fold. For these 10-fold CV runs, we utilized the set of fixed hyperparameters (such as learning rate and embedding dimensions) that were determined as optimal during our initial single-fold experiments, and each fold was trained for a number of epochs consistent with those initial experiments. Subsequently, the model was evaluated on the corresponding 10% original KDGene test set. These comprehensive 10-fold cross-validation results, alongside those for selected key baseline models under the same rigorous regimen, are detailed in the SM6: 10-fold Cross-Validation Results for Disease-Gene Prediction Task on DisGeNET.

Protein–Chemical Interaction task: This task aims to predict chemical entities that interact with a given protein. We utilize 23,074 STITCH interaction triples filtered for the top 100 most frequent proteins (Wang et al., 2024b), following the same 7:2:1 train-validation-test split. The model predicts chemical entities interacting with a given protein. For example, when presented with DRD2 (Dopamine Receptor D2), a key prediction would be acetylcholine (CIDm00000187). This specific interaction, annotated in the STITCH dataset as a protein-chemical relation, is biologically meaningful and reflects DRD2's established role in modulating cholinergic signaling. To address class imbalance from extensive negative sampling, supplementary samples are capped at 10,000 (disease-gene) and 10,000 (disease-drug).

Medical Ontology Reasoning task: This task is a form of Knowledge Graph Reasoning (KGR) and utilizes the comprehensive UMLS Terminology (Bodenreider, 2004). Following prior work (Zhang et al., 2023; Zhang and Yao, 2022), this terminology is pre-split into background, training, validation, and test sets. Within this KGR framework, the task is specifically formulated as a link prediction problem: given a head concept and a specific UMLS relation type, the model must predict the correct tail concept to complete the triplet. For instance, given the head UMLS concept Acquired_Abnormality and the relation Result_of, the model would aim to predict Phenomenon_or_Process as the tail; this specific prediction is ontologically justified because acquired medical conditions inherently result from various underlying phenomena or processes, thus forming the valid triplet (Acquired_Abnormality, Result_of, Phenomenon_or_Process). By concealing the tail entities of known relations during training and tasking the model with their prediction, reasoning is effectively transformed into a link prediction problem. This approach facilitates not only the completion of missing hierarchical links (e.g., predicting broader or narrower concepts using relations like "Isa" or "Part_of") and the discovery of implicit associations (e.g., through relations such as "Associated_with" or "Co-occurs_with"), but also the verification of consistency within medical domain knowledge.

(2) SM2: Gradient-Preserving Hard Selection Mechanism

The Gradient-Preserving Hard Selection Mechanism in BioGraphFusion selects a fixed-size set of K most relevant entities (Top-K nodes) from a candidate set $\mathcal{C}^{(\ell)}$ at each propagation layer ℓ. This focuses computational resources and directs information flow. A key challenge is performing this discrete Top-K selection differentiably for end-to-end training via gradient-based optimization.

The mechanism first computes continuous, soft relevance scores $\mathbf{s}_t$ for each candidate node $t \in \mathcal{C}^{(\ell)}$. During **training**, to achieve differentiable Top-K selection, BioGraphFusion employs the Gumbel-Softmax technique (Jang et al., 2017; Maddison et al., 2017). This allows the derivation of a "hard" binary selection mask $\mathbf{s}_t^{\text{hard}}$ (values near 0 or 1) from the soft scores $\mathbf{s}_t$, indicating the selected Top-K nodes $\mathcal{V}^{(\ell)}$:

$$\mathcal{V}^{(\ell)} = \text{TopK}\left(\mathbf{s}_t \mid t \in \mathcal{C}^{(\ell)}\right). \tag{1}$$

The Gumbel-Softmax ensures this process is amenable to gradient-based learning.

To preserve gradients through this discrete selection when updating node representations $\mathbf{h}_t^{(\ell)}$, the following strategy is used:

$$\mathbf{h}_t^{(\ell)} \leftarrow \mathbf{h}_t^{(\ell)} \cdot \left(\mathbf{s}_t^{\text{hard}} - \text{detach}(\mathbf{s}_t) + \mathbf{s}_t\right)$$

In the **Forward Pass**, this expression effectively multiplies $\mathbf{h}_t^{(\ell)}$ by the hard selection mask $\mathbf{s}_t^{\text{hard}}$. In the **Backward Pass**, the gradient with respect to the parameters generating $\mathbf{s}_t$ is equivalent to the gradient of $\mathbf{s}_t$ itself. This "straight-through estimator" variant allows discrete forward selection while using continuous soft scores for gradient estimation, preserving crucial gradient information.

During **inference**, differentiability is not needed, so a standard deterministic Top-K selection based on the highest soft scores $\mathbf{s}_t$ is used.

This mechanism is vital as it enables focused, discrete node selection while maintaining end-to-end differentiability for effective training. It combines interpretable hard selections with stable gradient-based learning, enhancing BioGraphFusion's ability to capture salient information for accurate knowledge graph completion and reasoning.

(3) SM3: Experimental Configuration and Hyperparameter Optimization

We implemented all experiments in Python using PyTorch v1.12.1 and PyTorch Geometric v2.0.9 on a single NVIDIA RTX 3090 GPU. Training time and GPU memory usage vary with the dataset and batch size. Hyperparameter tuning was performed over the following ranges (see Table 2): learning rate from $\{10^{-4}, 5\times10^{-4}, 10^{-3}, 5\times10^{-3}, 10^{-2}\}$; batch size from $\{4, 8, 16, 32\}$; embedding dimension D from $\{32, 48, 64, 80, 96\}$; selected entities count K from $\{100, 300, 500, 800, 1000\}$; fusion weight λ from $\{0.3, 0.4, 0.5, 0.6, 0.7, 0.8\}$; regularization coefficient γ from $\{0, 0.001, 0.01, 0.1\}$; and propagation steps ℓ from $\{4, 5, 6, 7, 8\}$. Other hyperparameters were set following the AdaProp configuration (Zhang et al., 2023). The optimal settings were selected based on the MRR metric evaluated on the validation set $\mathcal{F}_{\text{val}}$, with training capped at 100 epochs.

The code implementation reflecting these optimal hyperparameter settings is publicly available in our open-source repository.

(4) SM4: Evaluation Metrics

We evaluate our model's performance on the biomedical knowledge graph tasks using two standard ranking metrics: Mean Reciprocal Rank (MRR) and Hit@k, following established practices (Wang et al., 2024a; Zhang and Yao, 2022; Zhang

Table 2. Hyperparameter Settings for BioGraphFusion Experiments

Hyperparameters	Values
Learning Rate	$\{10^{-4}, 5 \times 10^{-4}, 10^{-3}, 5 \times 10^{-3}, 10^{-2}\}$
Batch Size	$\{4, 8, 16, 32\}$
Embedding Dimension D	$\{16, 32, 48, 64, 96\}$
Selected Entities Count K	$\{100, 300, 500, 800, 1000\}$
Fusion Weight λ	$\{0.3, 0.4, 0.5, 0.6, 0.7, 0.8\}$
Regularization Coefficient γ	$\{0, 0.001, 0.01, 0.1\}$
Propagation Steps ℓ	$\{4, 5, 6, 7, 8\}$

et al., 2023). These metrics are chosen to quantify both the overall ranking quality and the model's precision in retrieving the correct entity within the top-k predictions.

The Mean Reciprocal Rank (MRR) is defined as:

$$\text{MRR} = \frac{1}{|\mathcal{Q}|} \sum_{(q_e,q_r,q_a)\in\mathcal{Q}} \frac{1}{\text{rank}(q_e, q_r, q_a)}$$

where $\mathcal{Q}$ denotes the set of test queries in the form $(q_e, q_r, ?)$ and $\text{rank}(q_e, q_r, q_a)$ indicates the position of the correct tail entity q_a for the query $(q_e, q_r, ?)$ in a filtered ranking list. In the filtered setting, all known true triples (except for the target triple) are removed from the candidate list to ensure that only plausible negatives are considered. MRR provides an average measure of ranking quality across all test queries, with higher values indicating better overall performance.

The Hit@k metric measures the proportion of queries for which the correct entity appears within the top k predictions. It is computed as:

$$\text{Hit@}k = \frac{1}{|\mathcal{Q}|} \sum_{(q_e,q_r,q_a)\in\mathcal{Q}} \mathbb{I}\left(\text{rank}(q_e, q_r, q_a) \le k\right)$$

where $\mathbb{I}(\cdot)$ is the indicator function that returns 1 if the condition is true and 0 otherwise. This metric reflects the model's precision in retrieving the correct answer among the top predictions.

(5) SM5: Baseline Models and Implementation Protocols

Baseline Model Categories

To evaluate the performance of our framework in biological knowledge graph analysis, we benchmark against state-of-the-art methods from three major categories: Knowledge Embedding (KE) models, Graph Neural Network (GNN-based) approaches, and Ensemble methods:

For KE, we benchmark against: RotatE (Sun et al., 2019) modeling relations as complex space rotations; ComplEx (Trouillon et al., 2016) capturing asymmetric interactions via complex-valued embeddings; DistMult (Yang et al., 2014) using a simplified bilinear scoring function; CP-N3 (Lacroix et al., 2018) which enhances the Canonical Polyadic (CP) decomposition with N3 regularization; and KDGene (Wang et al., 2024a), a model specifically designed for disease-gene prediction using interactional tensor decomposition.

For GNNs, we compare with: pLogicNet (Qu and Tang, 2019) which integrates probabilistic logic with neural networks; CompGCN (Vashishth et al., 2019) that incorporates relation composition into Graph Convolutional Networks; DPMPN (Xu et al., 2019) employing a dynamic programming message passing network; AdaProp (Zhang et al., 2023) which learns adaptive propagation patterns for multi-hop reasoning; and RED-GNN (Zhang and Yao, 2022), a relational digraph-based GNN for knowledge graph reasoning.

For Ensemble methods, we include: KG-BERT (Yao et al., 2019), treating knowledge graph triples as textual sequences and fine-tuning pre-trained language models like BERT for triple plausibility scores; StAR (Wang et al., 2021), a hybrid model augmenting textual encoding with graph embedding techniques, utilizes a Siamese-style encoder and learns representations by employing both a deterministic classifier for semantic plausibility and a spatial distance measurement for structural relationships; and LASS (Shen et al., 2022), jointly embedding triplet natural language semantics and structure via pre-trained language model fine-tuning with a probabilistic loss.

Baseline Implementation and Evaluation Protocols

We utilize publicly available code from the original authors whenever possible, with download links provided in Table 3 to facilitate access to original implementations for further details and reproducibility.

To ensure a robust and fair comparison, we adopted a systematic approach to hyperparameter selection for all methods. For each baseline model, we began with the officially recommended or widely reported hyperparameter settings. We then carefully adjusted key parameters (such as batch size, learning rate, and training epochs) based on the characteristics of our biological knowledge graph datasets and preliminary results on a dedicated validation set, aiming to optimize each model's performance. Importantly, the final hyperparameters for all models were selected according to their performance (e.g., MRR) on the same validation set. This consistent validation protocol ensures that all models were evaluated under comparable conditions, enabling a fair and meaningful performance comparison.

Table 3. Baselines with URLs to download the codes provided by the respective authors.

Baselines	URLs
RotatE (Sun et al., 2019)	`https://github.com/DeepGraphLearning/KnowledgeGraphEmbedding`
ComplEx (Trouillon et al., 2016)	`https://github.com/ttrouill/complex`
DisMult (Yang et al., 2014)	`https://github.com/mana-ysh/knowledge-graph-embeddings`
CP-N3 (Lacroix et al., 2018)	`https://github.com/facebookresearch/kbc`
KDGene (Wang et al., 2024a)	`https://github.com/sienna-wxy/KDGene`
pLogicNet (Qu and Tang, 2019)	`https://github.com/DeepGraphLearning/pLogicNet`
CompGCN (Vashishth et al., 2019)	`https://github.com/malllabiisc/CompGCN`
DPMPN (Xu et al., 2019)	`https://github.com/anonymousauthor123/DPMPN`
AdaProp (Zhang et al., 2023)	`https://github.com/LARS-research/AdaProp`
RED-GNN (Zhang and Yao, 2022)	`https://github.com/LARS-research/RED-GNN`
KG-BERT (Yao et al., 2019)	`https://github.com/yao8839836/kg-bert`
LASS (Shen et al., 2022)	`https://github.com/jhshen95/LASS`
StAR (Wang et al., 2021)	`https://github.com/wangbo9719/StAR_KGC`

(6) SM6: 10-fold Cross-Validation Results for Disease-Gene Prediction Task on DisGeNET

To provide a fair and comprehensive evaluation of our BioGraphFusion model on the disease-gene prediction task, we employed a more rigorous 10-fold cross-validation experimental design. Since conducting complete 10-fold cross-validation for all baseline models would require substantial computational resources and time, we selected representative models that performed most competitively in each category from Table 1 in the main manuscript (KDGene representing the KE category, RED-GNN representing the GNN category, and StAR representing the ensemble learning category). We then ran experiments based on their publicly available code under the unified 10-fold cross-validation setting.

Table 4 presents the detailed performance of each model under 10-fold cross-validation, with results reported as “Mean ± Standard Deviation” to provide a more comprehensive reflection of model performance and stability. As demonstrated in the table, BioGraphFusion consistently outperforms other baseline methods across all three key metrics: MRR (0.436±0.014), Hit@1 (0.382±0.007), and Hit@10 (0.537±0.020). The reasonable distribution of standard deviation values further reflects the model’s stability and robustness across different data splits.

Table 4. Cross-validation results for Disease-Gene Prediction on the DisGeNET dataset. All models were evaluated using the original 10-fold splits defined by KDGene (Wang et al., 2024a). Results are reported as mean ± standard deviation across the 10 folds.

**Type**	**Model**	**MRR**	**Hit@1**	**Hit@10**
KE	KDGene (Wang et al., 2024a)	0.378 ± 0.016	0.315 ± 0.009	0.518 ± 0.017
GNN	RED-GNN (Zhang and Yao, 2022)	0.394 ± 0.012	0.338 ± 0.008	0.472 ± 0.018
Ensemble	StAR (Wang et al., 2021)	0.241 ± 0.015	0.185 ± 0.010	0.354 ± 0.021
Proposed	BioGraphFusion (ours)	0.436 ± 0.014	0.382 ± 0.007	0.537 ± 0.020

Regarding standard deviation, we observe distinct patterns across different methods: KDGene shows a relatively higher standard deviation in MRR (0.016), which may reflect the sensitivity of knowledge embedding methods when handling different data splits; RED-GNN demonstrates a moderate level of standard deviation (e.g., 0.012 for MRR), indicating that graph structure-based methods maintain relative stability in local structure modeling; while StAR’s higher standard deviation in Hit@10 (0.021) might stem from its ensemble approach responding differently to variations in data distribution. In comparison, BioGraphFusion maintains reasonable and relatively low standard deviations across most metrics (0.014 for MRR, 0.007 for Hit@1), particularly demonstrating excellent performance on the strict Hit@1 metric, which fully proves that our approach not only delivers superior performance but also exhibits good stability, capable of providing reliable predictions under various data conditions.

(7) SM7: Computational Efficiency Analysis and Predictive Accuracy on UMLS Dataset

To provide a comprehensive assessment of the trade-off between computational efficiency and predictive accuracy, we present a comparative analysis of BioGraphFusion’s inference performance relative to representative baseline models on the UMLS dataset. Inference times were averaged over five independent runs, all conducted under identical hardware and software conditions (single NVIDIA RTX 3090 GPU, PyTorch 1.12.1, Python 3.10.14).

For this comparative analysis, we selected high-performing and representative models from three main categories: Knowledge Embedding (KE) models (specifically RotatE and ComplEx), Graph Neural Networks (GNNs) (AdaProp and RED-GNN), and LM-based Ensemble approaches (LASS and StAR). Figure 2 provides a visual summary, plotting the Mean Reciprocal Rank (MRR) on the UMLS test set against the corresponding inference time (in seconds) for BioGraphFusion and these selected baselines.

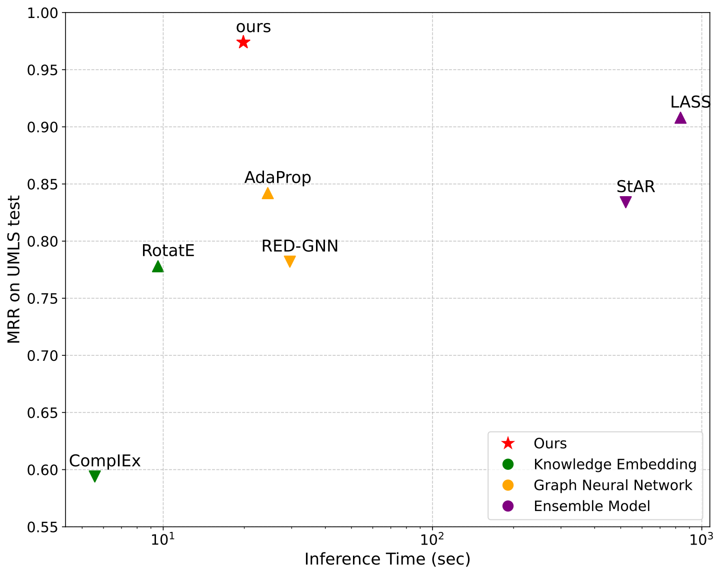

Fig. 2: Comparative analysis of computational efficiency and predictive accuracy on the UMLS dataset (inference time measured on NVIDIA RTX 3090 GPU). The scatter plot displays the Mean Reciprocal Rank (MRR) versus inference time (seconds) for BioGraphFusion (ours) and representative baseline models: Knowledge Embedding (RotatE, ComplEx), Graph Neural Network (AdaProp, RED-GNN), and Ensemble (LASS, StAR). Inference times are averaged over five runs using each model's optimal hyperparameters. This figure illustrates the balance between predictive performance and computational efficiency across different modeling approaches.

As depicted in Figure 2, on the UMLS dataset, BioGraphFusion achieves a high MRR of 0.974 with an inference time of approximately 19.84 seconds. This positions BioGraphFusion effectively within the performance-efficiency landscape. Its inference efficiency is notably strong, particularly when compared to the other GNNs evaluated; for instance, it is faster than both AdaProp (inference time: 24.45s, MRR: 0.842) and RED-GNN (inference time: 29.54s, MRR: 0.782), while also achieving a higher MRR. Relative to traditional KE methods, such as RotatE (inference time: 9.56s, MRR: 0.778) and ComplEx (inference time: 5.57s, MRR: 0.594), which are generally faster due to their simpler architectures, BioGraphFusion demonstrates a substantial improvement in MRR for a moderate increase in inference time. Furthermore, BioGraphFusion is significantly more efficient in its inference phase than the typically more computationally demanding LM-based ensemble systems such as LASS (inference time: 833.89s, MRR: 0.908) and StAR (inference time: 522.0s, MRR: 0.834). BioGraphFusion not only boasts a much shorter inference time than these LM-based models but also achieves a higher MRR than both.

BioGraphFusion's favorable balance between high performance and efficient inference appears to stem from its sophisticated design that addresses key limitations of other paradigms. The framework's use of global semantic guidance, derived from tensor decomposition, likely enables more direct and interpretable pathfinding during reasoning. This global guidance can inform BioGraphFusion's query-guided subgraph construction, allowing it to selectively build more focused and semantically relevant subgraphs. This potentially streamlines the process compared to GNNs that might rely on more computationally intensive or exploratory subgraph generation strategies when lacking such strong initial global priors. Moreover, unlike some LM-based approaches that may not fully or efficiently integrate dynamic semantic insights with evolving structural graph updates during multi-hop reasoning, or traditional KE techniques that might oversimplify relational patterns and overlook critical graph structure, BioGraphFusion is designed to foster a deeper, adaptive interaction between semantic context and structural information. While some simpler KE methods offer faster raw inference speeds, BioGraphFusion strikes a compelling balance: achieving efficient processing within an expressive framework that supports complex, stable, and ultimately more effective knowledge graph reasoning by robustly modeling and leveraging the crucial interplay between semantics and structure. This demonstrates that BioGraphFusion can attain state-of-the-art predictive accuracy without incurring the prohibitive computational overheads observed in some other advanced, particularly LM-based, models.

(8) SM8: Regularization Studies and LSTM Validation

Validation of LSTM for Contextual Relation Refinement

To empirically validate our architectural choice of Long Short-Term Memory (LSTM) networks for the Contextual Relation Refinement Module, we conducted comprehensive comparative experiments where this critical component was systematically replaced with alternative neural architectures. As detailed in Table 5, the LSTM-based configuration of BioGraphFusion consistently demonstrated superior or highly competitive performance across all three evaluated datasets. For instance, on the Disease-Gene Prediction task, it achieved notable metrics (MRR 0.429, Hit@1 0.377, Hit@10 0.529), outperforming RNN and GRU variants. This trend of superior contextual modeling was similarly observed in the other biomedical tasks, reinforcing its general applicability.

This performance advantage directly stems from LSTM's architectural design, which proves exceptionally well-suited for biomedical knowledge graphs where relation semantics are highly entity-dependent. While RNN and GRU demonstrated diminished efficacy due to their simpler memory mechanisms, LSTM's sophisticated structure provides multiple critical advantages through its tripartite gating system and dedicated memory cell. The forget gate selectively discards irrelevant aspects of prior relation states, the input gate regulates incorporation of entity-derived information, and the output gate determines which aspects propagate as the new relation embedding. This architecture enables LSTM to effectively preserve long-term dependencies and perform fine-grained contextual adjustments that RNN and GRU cannot achieve with their simplified structures, particularly in complex biomedical contexts where relation meanings shift substantially based on specific entity pairs.

Even when compared to more complex architectures like Echo State Networks and Temporal Convolutional Networks, the LSTM-based configuration demonstrated superior performance. ESNs' static reservoir design inherently limits adaptive memory control compared to LSTM's flexible gating. Similarly, while TCNs excel at sequence-level pattern processing, they are less aligned with our task's demand for targeted entity-specific relation modulation. The Transformer architecture, while undeniably powerful for capturing global dependencies in many sequence processing tasks, showed inferior performance for our particular module focused on iterative, entity-specific refinement of individual relation embeddings when compared to our LSTM-based configuration.

LSTM's inherent support for stateful updates, where output depends on both previous state and current inputs, perfectly aligns with our goal of iteratively refining relation embeddings based on contextual entity information. This allows the model to learn complex, adaptive mappings that tailor relation representations to specific entity pairs—essential for modeling the intricate biological processes whose interpretation varies significantly with participating molecular entities.

These empirical findings and theoretical considerations robustly affirm LSTM as the optimal architecture for contextual relation refinement in BioGraphFusion, highlighting the importance of aligning neural architecture characteristics with specific task demands in complex biomedical domains.

Table 5. Comparative Performance of BioGraphFusion Variants: Validating LSTM for Contextual Relation Refinement and Assessing the Impact of N3 Regularization. Best results are shown in **bold**, and second-best results are underlined.

BioGraphFusion Model variants	Disease-Gene Prediction			Protein-Chemical Interaction			Medical Ontology Reasoning		
	MRR	Hit@1	Hit@10	MRR	Hit@1	Hit@10	MRR	Hit@1	Hit@10
w/ RNN	0.387	0.332	0.492	0.654	0.603	0.752	0.942	0.924	0.982
w/ GRU	0.386	0.330	0.495	0.659	0.608	0.755	0.951	0.936	0.984
w/ ESN	0.407	0.344	<u>0.521</u>	0.683	0.632	0.778	0.965	0.947	<u>0.990</u>
w/ TCN	0.404	0.353	0.515	0.689	0.639	0.782	<u>0.969</u>	<u>0.952</u>	0.988
w/ Transformer	0.383	0.321	0.500	0.671	0.621	0.765	0.953	0.934	0.985
w/ L1	0.409	0.358	0.518	0.687	0.637	0.780	0.961	0.945	0.987
w/ L2	<u>0.412</u>	<u>0.364</u>	0.520	<u>0.694</u>	<u>0.645</u>	<u>0.788</u>	0.967	0.950	0.989
w/o N3	0.395	0.338	0.509	0.676	0.626	0.769	0.955	0.937	0.986
**Ours (w/ LSTM & N3)**	**0.429**	**0.377**	**0.529**	**0.702**	**0.657**	**0.795**	**0.974**	**0.963**	**0.991**

Analysis of N3 Regularization Impact

This section assesses the role of N3 regularization within the BioGraphFusion framework, with a particular focus on determining whether it acts as a confounding factor for the model's performance improvements. N3 regularization is employed in BioGraphFusion, following established approaches such as CP-N3 (Lacroix et al., 2018), to operate on the CP embeddings, specifically $\mathbf{e}_{q_e}$, $\mathbf{e}^{\ell}_{q_r}$, and $\mathbf{e}_{q_a}$. The technique targets the sum of cubes of embedding magnitudes. This form of regularization is intended to mitigate overfitting and enhance the model's ability to learn from complex biological knowledge graphs. We aim to demonstrate that BioGraphFusion's core architectural innovations provide substantial benefits independently, and that N3 regularization serves as a well-suited, complementary component rather than the sole driver of high performance.

To dissect the precise influence of N3 regularization, we compare our full BioGraphFusion model against several alternative configurations, as detailed in Table 5. These include variants of BioGraphFusion that incorporate standard L1 regularization (L1) and standard L2 regularization (L2), both applied to the same CP embeddings. Additionally, we evaluate a BioGraphFusion variant from which N3 regularization has been entirely removed. This comparative analysis allows for a clear delineation of N3's contribution relative to other regularization methods and its overall necessity.

This analysis first evaluated if BioGraphFusion's foundational architecture performs strongly without the specific N3 regularization. As shown in Table 5, on the Disease-Gene Prediction task, BioGraphFusion with L2 regularization (MRR 0.412) and L1 regularization (MRR 0.409) both surpassed the strongest competing baseline model. Similar strong performance with L1 and L2 regularization, relative to baselines, was observed across the other datasets as well. These results highlight that BioGraphFusion's primary efficacy stems from its core architectural innovations: the establishment of a global semantic foundation through Canonical Polyadic (CP) decomposition, dynamic structural reasoning actively guided by these initial embeddings, and advanced, context-aware relation refinement using LSTMs. This establishes that

the model's competitive edge is rooted in this inherent design, which fosters a synergistic interplay between semantic understanding and structural learning, rather than depending solely on N3 regularization, thus confirming its robust foundational design.

While the core architecture is strong, regularization choice significantly impacts final performance. Our BioGraphFusion model with N3 regularization achieved the highest scores, notably outperforming its L2, L1, and non-regularized counterparts. This consistent performance hierarchy indicates that although any regularization is beneficial over none, N3 offers a distinct advantage over conventional L1 and L2 methods for this model.

The superior performance of N3 regularization suggests that its specific mechanism, which penalizes the (l_3)-norm of embedding magnitudes, is particularly well-aligned with the characteristics of the CP embeddings used and the demands of biological knowledge graph tasks. This type of regularization may offer a more suitable inductive bias for navigating the complex and often sparse relationships prevalent in such data. Consequently, it appears to lead to more effective prevention of overfitting and improved generalization capabilities compared to L1 or L2 norms in this specific context.

In essence, BioGraphFusion's primary effectiveness is rooted in its strong and innovative core architecture, which delivers competitive results independently. The N3 regularization then acts as a significant and synergistic enhancement, further elevating the model's performance to a state-of-the-art level.

(9) SM9: t-SNE Visualizations and Embedding Analysis with Baseline Models

To evaluate the semantic quality of embeddings learned by BioGraphFusion relative to other approaches, we conducted a comparative t-SNE visualization analysis (Fig. 3). This analysis focuses on protein embeddings from the Protein-Chemical Interaction task, specifically visualizing those associated with 10 chemical compounds (PubChem CIDs), each linked to 50–100 interacting proteins. BioGraphFusion's embeddings are compared against those from three representative baselines: RotatE (knowledge graph embedding) (Sun et al., 2019), RED-GNN (GNN-based) (Zhang and Yao, 2022), and StAR (ensemble text and structure) (Wang et al., 2021).

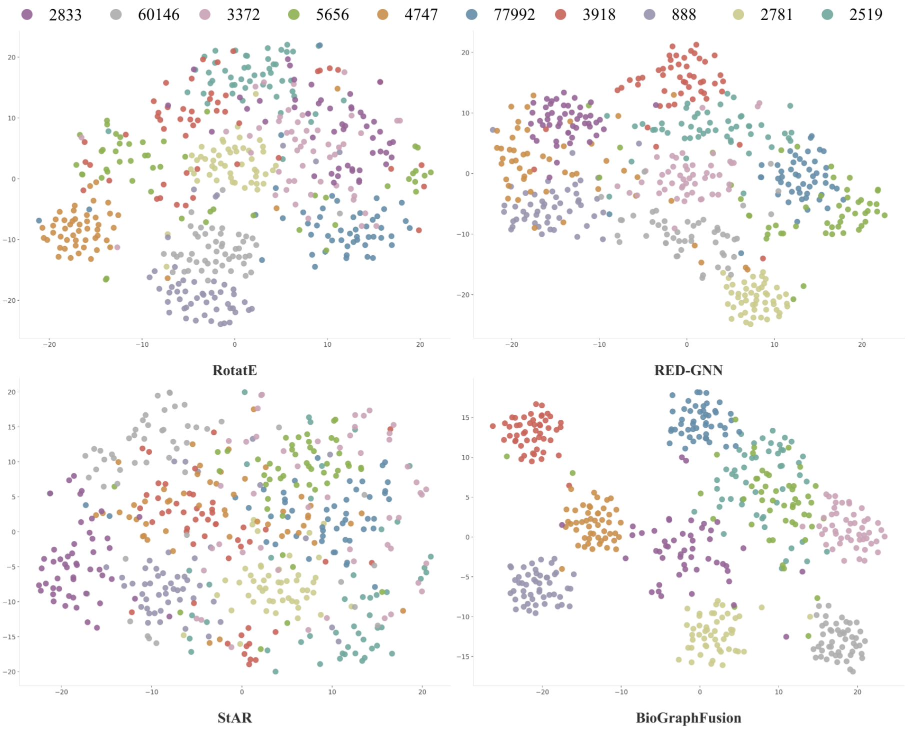

Fig. 3: t-SNE visualization of protein embeddings. Each subfigure shares the same proteins and each color represents proteins interacting with the same chemical compound, labeled by PubChem CID.

Protein embeddings for each method are obtained from their final learned representations. For GNN-based models like RED-GNN and our BioGraphFusion, these are typically generated after message propagation phases. For KE models like RotatE, embeddings are the direct output of the training process that optimizes a scoring function for triples. For StAR, embeddings are derived from its framework that integrates textual encoding with structural information. These embeddings are then reduced to two dimensions for visualization. The t-SNE plot corresponding to the BioGraphFusion model (shown in Fig. 3) yields tightly clustered and semantically coherent protein embeddings, indicating that the integration of CP decomposition-derived initial embeddings and LSTM-driven relation updates effectively shapes the representations to capture fine-grained interaction contexts.

In contrast, the selected baseline methods, as depicted in their respective t-SNE visualizations (Fig. 3), tend to exhibit more diffuse embedding clusters with notable inter-group overlap. RotatE's visualization shows that while this method is effective at modeling relational patterns through rotations in complex space, its representations (learned primarily based on existing triple structures) may not always group entities optimally based on the specific, nuanced semantic context of diverse protein-chemical interactions if such distinct contexts are not explicitly and sufficiently captured by distinct relational paths. This can potentially lead to less distinct clusters for proteins interacting with different chemicals. For RED-GNN, its strength lies in capturing structural information and multi-hop relations via its relational digraph structure and query-attentive message passing mechanisms. While proficient in reasoning over graph structures, its inherent semantic differentiation for entities primarily depends on the learned relation embeddings and topological neighborhood information. In our experimental setup, the input features to RED-GNN did not contain rich initial semantic information beyond identifiers, and its GNN architecture primarily focused on propagating structural signals. Consequently, this resulted in broader, less separated clusters in its t-SNE plot compared to models that more deeply integrate explicit semantic features or leverage descriptive inputs.

StAR's t-SNE visualization is also considered. This model is designed as a hybrid approach augmenting textual encoding of triples with graph embedding techniques, using a Siamese-style textual encoder. Its ability to form distinct semantic clusters is significantly influenced by the availability and richness of descriptive text for proteins and chemicals. In our experimental setup for the PCI task, to maintain consistency in the input features across all compared methods, detailed textual descriptions for entities were not incorporated for any model; entities were primarily represented by their identifiers or brief names. Consequently, StAR's powerful textual encoding capabilities, which rely on such rich descriptions, were not fully leveraged in this setting. The model, therefore, likely relied more heavily on its structural learning components or the default textual interpretation of these basic identifiers. This absence of detailed descriptive semantic input may have contributed to less defined separations between protein groups in the visualization, as the model might not have captured the subtle distinguishing features necessary for tight, well-separated clustering based on their specific chemical interactions.

(10) SM10: Hyperparameter Sensitivity Analysis on the Disease-Gene Prediction Task

To examine how key hyperparameters influence the predictive performance of BioGraphFusion, we conducted a sensitivity analysis on the disease-gene prediction task. We systematically varied batch size, embedding dimension D, fusion weight λ, and the number of propagation steps ℓ, observing their effects on ranking metrics such as MRR, Hit@1, and Hit@10. The analysis helps identify optimal parameter settings that maximize model effectiveness while maintaining stability across different configurations. The following sections detail the observed trends for each hyperparameter, with results visualized in Fig. 4.

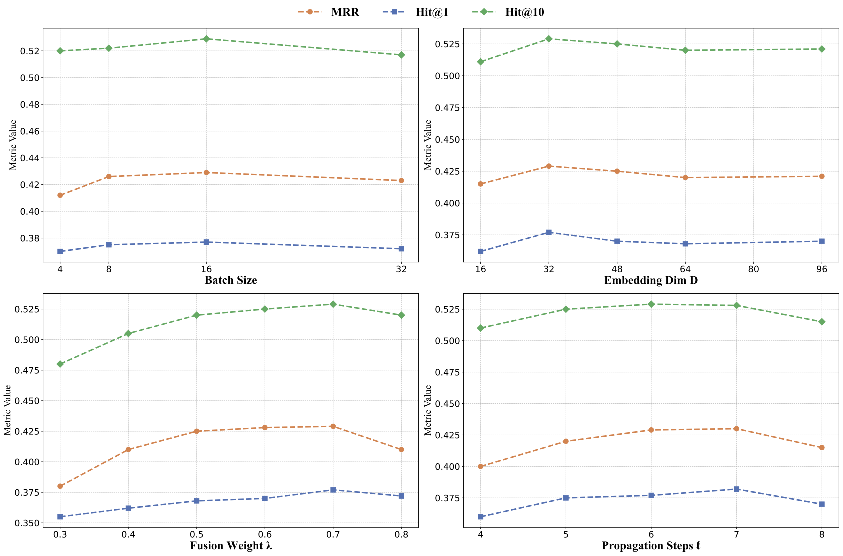

Fig. 4: BioGraphFusion underwent a hyperparameter sensitivity analysis to evaluate the influence of batch size, embedding dimension (D), fusion weight λ, and propagation steps (ℓ) on the resulting performance, as measured by MRR, Hit@1, and Hit@10.

Impact of Batch Size

The selection of an appropriate batch size is crucial for balancing model performance and training efficiency. Our search space for this hyperparameter was guided by the common practice of using power-of-2 batch sizes for computational

efficiency and the practical constraints of GPU memory. Preliminary tests indicated that batch sizes exceeding 50 were unfeasible for our single-GPU (NVIDIA RTX 3090) training setup. Consequently, we established the experimental batch size range for performance evaluation as [4, 8, 16, 32].

We evaluated performance metrics (MRR, Hit@1, and Hit@10) across this range. Optimal performance was achieved with a batch size of 16, while a batch size of 8 also yielded strong and comparable results. A decline in performance was observed for the smallest batch size tested (4) and for the largest (32), relative to these optima. The reduced performance at a batch size of 4 is consistent with the known challenge of less stable gradient estimations, which can hinder effective convergence. These findings indicate that batch sizes of 8 and 16 offer the most favorable performance characteristics for this task.

Notably, smaller batch sizes inherently increase training duration. This increase is attributable not only to the higher frequency of parameter updates but also to the potential underutilization of parallel processing capabilities in hardware accelerators. Therefore, while our results provide guidance on optimal performance, we recommend that users adjust the batch size according to their available computational resources and desired training speed. This flexible approach ensures that model training remains practical and can be tailored to individual operational contexts, balancing performance with computational feasibility.

Impact of Embedding Dimension D

We experimented with embedding dimensions ranging from 16 to 96. Performance shows a trend of initial improvement followed by a plateau or gradual decline, with the best results observed at dimension $D = 32$. This phenomenon indicates that a moderate embedding dimension is sufficient to capture the necessary semantic details without incurring over-parameterization or redundancy.

Our findings emphasize the critical role of carefully tuning the embedding dimensions. Not only do they balance model expressiveness and robustness, but they also play a fundamental part in capturing the inherent semantic structure of the biomedical knowledge graph. Given that the CP decomposition is specifically designed to unveil latent semantic patterns in multi-relational data, the chosen embedding dimension directly affects how effectively these semantic details are preserved in the learned representations. Therefore, determining an optimal embedding size is essential to fully realizing the potential of our model, ensuring that the resulting embeddings are compact enough to faithfully reflect the global semantic structure hidden in the data.

Impact of Fusion Weight λ

The fusion weight λ is a crucial hyperparameter in BioGraphFusion, determining the balance between structural propagation and global semantic embeddings within the scoring function. A well-calibrated λ is essential for optimizing model performance across different datasets and biomedical knowledge graph structures. To systematically investigate its impact, we conducted experiments by varying λ over the set $\{0.3, 0.4, 0.5, 0.6, 0.7, 0.8\}$, evaluating model effectiveness using MRR, Hit@1, and Hit@10.

Our results reveal a clear trend: performance, as measured by MRR, Hit@1, and Hit@10, consistently improves as λ increases from 0.3, peaking at $\lambda = 0.7$ (MRR 0.429, Hit@1 0.377, Hit@10 0.529). Beyond this point, increasing λ to 0.8 leads to a slight performance decline. This observed peak at $\lambda = 0.7$ suggests an optimal calibration where the model achieves a harmonious integration of its diverse information sources—primarily structural patterns and semantic knowledge. At this value, these components achieve a synergy, contributing in the most balanced and effective manner to the model's final predictions. The subsequent performance dip beyond $\lambda = 0.7$ indicates that higher values may disrupt this calibrated balance, leading to a less complementary interplay of the model's informational components and, consequently, suboptimal generalization.

Consequently, the parameter λ in our scoring function, $\tilde{f}(q_e, q_r, q_a) = \lambda f(q_e, q_r, q_a) + (1 - \lambda)\phi(q_e, q_r, q_a)$, should not be misconstrued as a simple toggle weighting 'pure' Graph Structure Propagation (GSP) against 'pure' Knowledge Embedding (KE). Instead, λ fine-tunes the contributions of two distinct yet deeply intertwined components to the final score: $f(q_e, q_r, q_a)$, which represents the output of our GSP module (itself initialized and guided by KE), and $\phi(q_e, q_r, q_a)$, which is the direct score from our tensor decomposition-based KE module.

Given this inherent coupling—where KE directly informs the GSP process that yields f—KE's influence is integral to both terms, albeit differently. While ϕ offers a direct semantic score, f provides structural insights shaped by KE. Thus, λ balances their ultimate contributions to the score, but the underlying structural information (channeled through f) and the overarching semantic context (from ϕ and also embedded within f) are both fundamental to the model's predictive capabilities. Optimizing λ is therefore aimed at an ideal balance between global semantic knowledge from embeddings and the information gathered through semantically-guided graph propagation. This crucial balance ensures global semantics effectively steer graph propagation, helping to capture fine-grained, local interactions. A well-calibrated model can then comprehensively represent diverse relationships within biomedical graphs, ultimately yielding robust and accurate predictive scores.

Impact of Propagation Steps ℓ

We evaluated the number of propagation steps ℓ over $\{4, 5, 6, 7, 8\}$. Performance improves with increasing ℓ, peaking at $\ell = 6$ (with MRR of 0.429, Hit@1 of 0.377, and Hit@10 of 0.529), and then declines at $\ell = 8$. This suggests that an optimal propagation depth exists: too few steps limit the model's ability to capture extended neighborhood information, whereas too many steps lead to over-smoothing, causing node representations to become overly similar and less discriminative.

The results indicate that a propagation depth of 6 best balances information aggregation and preservation of distinct node features.

The above analysis is specifically for the Disease-Gene dataset. Nevertheless, we speculate that a similar trend regarding the parameter ℓ will be observed on other datasets, namely the existence of an optimal propagation depth that balances information propagation and over-smoothing. However, the specific numerical value of the optimal ℓ may vary depending on the characteristics of the practical dataset.

(11) SM11: Protein-Protein Interaction Network Analysis of Known and Predicted Melanoma-Associated Genes

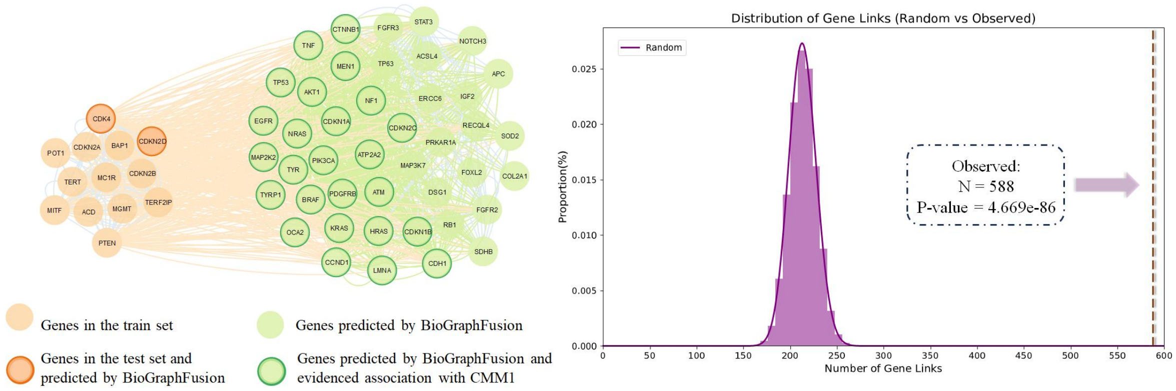

Fig. 5: Link visualization of known and predicted genes for melanoma on the PPI network. & For melanoma, the observed number of network links is significantly larger than the random control (P = 4.669E-86, binomial test)

To assess the functional relevance of the genes identified by BioGraphFusion, we analyzed the connectivity patterns within the Protein-Protein Interaction (PPI) network. By comparing the observed number of interactions between known melanoma-associated genes and predicted candidates with a randomly expected baseline, we evaluated the statistical significance of these connections using a binomial test. The results clearly indicate that the observed connectivity is far greater than expected by chance.

The resulting PPI network (Fig. 5) reveals 588 actual interactions among known and predicted genes, substantially exceeding the expected 213.17 connections under a random model (P = 4.669E-86, binomial test). This high degree of interconnectivity suggests that the predicted genes are functionally linked to known melanoma-associated genes, potentially sharing key pathways involved in disease progression. These findings demonstrate the robustness and reliability of the BioGraphFusion predictive framework, reinforcing its potential as a powerful tool for uncovering novel candidate genes with functional relevance. The model's ability to integrate diverse biological data and accurately predict gene interactions highlights its utility in advancing melanoma research and guiding further experimental validation.

References

O. Bodenreider. The unified medical language system (umls): integrating biomedical terminology. *Nucleic Acids Research*, 32:D267–D270, 2004.

E. Jang, S. Gu, and B. Poole. Categorical reparameterization with gumbel-softmax. *stat*, 1050:5, 2017.

M. Kuhn, I. Letunic, L. J. Jensen, and P. Bork. The sider database of drugs and side effects. *Nucleic acids research*, 44 (D1):D1075–D1079, 2016.

T. Lacroix, N. Usunier, and G. Obozinski. Canonical tensor decomposition for knowledge base completion. In J. Dy and A. Krause, editors, *Proceedings of the 35th International Conference on Machine Learning*, volume 80 of *Proceedings of Machine Learning Research*, pages 2863–2872. PMLR, 2018.

C. Maddison, A. Mnih, and Y. Teh. The concrete distribution: A continuous relaxation of discrete random variables. In *Proceedings of the international conference on learning Representations*. International Conference on Learning Representations, 2017.

M. Qu and J. Tang. Probabilistic logic neural networks for reasoning. *Advances in neural information processing systems*, 32, 2019.

J. Shen, C. Wang, L. Gong, and D. Song. Joint language semantic and structure embedding for knowledge graph completion. In *Proceedings of the 29th International Conference on Computational Linguistics*, pages 1965–1978, 2022.

Z. Sun, Z.-H. Deng, J.-Y. Nie, and J. Tang. Rotate: Knowledge graph embedding by relational rotation in complex space. *arXiv preprint arXiv:1902.10197*, 2019.

D. Szklarczyk, A. Santos, C. Von Mering, L. J. Jensen, P. Bork, and M. Kuhn. Stitch 5: augmenting protein–chemical interaction networks with tissue and affinity data. *Nucleic acids research*, 44(D1):D380–D384, 2016.

T. Trouillon, J. Welbl, S. Riedel, É. Gaussier, and G. Bouchard. Complex embeddings for simple link prediction. pages 2071–2080, 2016.

S. Vashishth, S. Sanyal, V. Nitin, and P. Talukdar. Composition-based multi-relational graph convolutional networks. *arXiv preprint arXiv:1911.03082*, 2019.

B. Wang, T. Shen, G. Long, T. Zhou, Y. Wang, and Y. Chang. Structure-augmented text representation learning for efficient knowledge graph completion. In *Proceedings of the Web Conference 2021*, pages 1737–1748, 2021.

X. Wang, K. Yang, T. Jia, F. Gu, C. Wang, K. Xu, Z. Shu, J. Xia, Q. Zhu, and X. Zhou. Kdgene: knowledge graph completion for disease gene prediction using interactional tensor decomposition. *Briefings in Bioinformatics*, 25(3): bbae161, 2024a.

Y. Wang, Z. Yang, and Q. Yao. Accurate and interpretable drug-drug interaction prediction enabled by knowledge subgraph learning. *Communications Medicine*, 4(1):59, 2024b.

X. Xu, W. Feng, Y. Jiang, X. Xie, Z. Sun, and Z.-H. Deng. Dynamically pruned message passing networks for large-scale knowledge graph reasoning. *arXiv preprint arXiv:1909.11334*, 2019.

B. Yang, W.-t. Yih, X. He, J. Gao, and L. Deng. Embedding entities and relations for learning and inference in knowledge bases. *arXiv preprint arXiv:1412.6575*, 2014.

L. Yao, C. Mao, and Y. Luo. Kg-bert: Bert for knowledge graph completion. *arXiv preprint arXiv:1909.03193*, 2019.

Y. Zhang and Q. Yao. Knowledge graph reasoning with relational digraph. In *Proceedings of the ACM Web Conference 2022*, page 912–924, New York, NY, USA, 2022. Association for Computing Machinery.

Y. Zhang, Z. Zhou, Q. Yao, X. Chu, and B. Han. Adaprop: Learning adaptive propagation for graph neural network based knowledge graph reasoning. In *Proceedings of the 29th ACM SIGKDD Conference on Knowledge Discovery and Data Mining*, pages 3446–3457, 2023.
